# Little evidence for cultural differences in automatic imitation

**DOI:** 10.1038/s44271-026-00517-5

**Published:** 2026-10-14

**Authors:** Oliver Genschow, Mareike Westfal, Angela R. Dorrough, Emiel Cracco, Tobias Wingen, Jan Crusius

**Affiliations:** 1 School of Management and Technology, Leuphana University Lüneburg, Lüneburg, Germany; 2 Social Cognition Center Cologne, University of Cologne, Cologne, Germany; 3 Faculty of Psychology, FernUniversität in Hagen, Hagen, Germany; 4 Department of Experimental Clinical and Health Psychology, Ghent University, Ghent, Belgium; 5 Institute for Psychology, University of Greifswald, Greifswald, Germany

**Keywords:** Human behaviour, Human behaviour

## Abstract

Individuals tend to automatically imitate others’ actions. Such imitative behavior is often assumed to be a universal phenomenon. However, most prior research has focused on Western, Educated, Industrialized, Rich, and Democratic (WEIRD) populations, leaving open whether people across different cultures imitate others in a similar manner. Ideomotor theory and the theory of objective self-awareness suggest that imitation is influenced by the extent to which individuals focus on the self versus others. Since cultural background strongly shapes self-construal—particularly along the collectivism-individualism dimension—cultural context may play a key role in shaping imitative behavior. To test this prediction, we examined automatic imitation in representative samples across 22 culturally diverse nations varying in collectivism and individualism. Consistent with prior findings, we replicated the basic automatic imitation effect across all countries but found no compelling evidence for cultural differences in automatic imitation. These findings hint to the possibility that automatic imitation is a universal phenomenon with little room for strong modulations by culture.

## Abstract (web summary)

This study investigates whether automatic imitation differs across representative samples from 22 culturally diverse nations. We find no credible evidence for cultural differences, supporting the idea that automatic imitation is a robust and widespread human tendency.

## Introduction

People tend to automatically imitate each other^1,2^. Research has shown that such imitative behavior serves an important social function, as it bonds humans together by creating feelings of affiliation and mutual understanding^3,4^. For example, imitation facilitates social interactions^5^ and increases liking for each other^5–7^—to name just a few examples.

It is often assumed that automatic imitation is a universal phenomenon^1,2^. However, since research on imitative behavior has mainly been conducted in Western, Educated, Industrialized, Rich, and Democratic (WEIRD)^8^ societies, an open question is how universal the phenomenon of imitation actually is and whether people from different cultures vary in how they imitate others. In particular, the question arises whether there are cultural differences in the underlying processes of automatic imitation. To shed light onto this question and further the understanding of the underlying mechanisms of imitative behavior, we investigated automatic imitation in representative samples in terms of age and gender across 22 different nations varying in the cultural dimension of collectivism/individualism.

### Measuring automatic imitation

A classic task for measuring imitative behavior is the imitation-inhibition task^9–11^. In this stimulus-response (S-R) compatibility task, participants respond to an imperative cue (e.g., number “1” or “2”) presented on the computer screen by lifting either the index or the middle finger. At the same time, they observe another person’s hand lifting either the same finger (congruent movement) or a different finger (incongruent movement). The logic of the task is that the instruction to engage in a different action than another person interferes with individuals’ automatic tendency to imitate, and as a consequence, slows down their response. Conversely, being instructed to respond with the same action as another person corresponds with individuals’ automatic tendency to imitate others, which speeds up their response. In line with this reasoning, the typical finding in the imitation-inhibition task is a congruency effect: participants respond faster and more accurately in congruent than incongruent trials^11^. Previous studies have shown that the imitation-inhibition task is a reliable^12^ as well as a valid and robust^13^ measure of automatic imitation.

In principle, two different components account for the classical congruency effect: Imitative compatibility and spatial compatibility. Imitative compatibility means that an action is facilitated when seeing another person engaging in the same movement (e.g., lifting) that involves the same effector (e.g., index finger). Spatial compatibility is present when an observed action spatially aligns with the executed action, such as when seeing a finger movement on the left side of the screen while performing a movement on the left side. Previous research^11^ has demonstrated that automatic imitation is mainly driven by imitative compatibility but also influenced by spatial compatibility. By explaining how stimulus and response dimensions in typical S-R tasks overlap, Kornblum et al.^14^ argue that responses speed up the more dimensions of a response overlap with the presented stimulus. In line with this view, imitative responses are faster when the movements are spatially aligned^11^ suggesting that the spatial dimension of a movement is an important aspect of automatic imitation.

### Processes underlying automatic imitation and relation to culture

According to ideomotor theory^15–17^, actions are represented as an image of its produced sensory feedback. As a consequence, repeatedly linking the sensory perception of another person’s action with performing that action oneself may lead to a motoric self-other overlap between one’s own motor intentions and those of others, due to the reinforcement of shared motor representations^18,19^. The idea that the observation of an action and the execution of the same action are based on shared motor plans has been confirmed by numerous studies including behavioral^9,10,20,21^, fMRI^22,23^, and motor TMS studies^24,25^, as well as single-cell recordings in monkeys^26^ and humans^27^. Previous research indicates that these shared representations serve as a foundation for processing information about both oneself and others, indicating that the brain inherently integrates self and other^28^.

Since self-other integration plays an important role in automatic imitation, the question arises whether focusing on the self, rather than on others, would reduce people’s automatic tendency to imitate. Based on the theory of objective self-awareness^29^, it has been proposed that when individuals are self-focused, the influence of perception on behavior can be altered^30^, because self-focus may enhance the execution of actions aligned with the self, rather than those linked to others. In line with this notion, some experiments found that when people are instructed to focus more strongly on the self, as compared to others, they imitate others’ movements less strongly^31–35^. It is important to note though that these findings should be considered as preliminary evidence for the idea that higher-order processes involved in self-other focus influence imitative behavior, as the literature produced mixed results^36^.

The degree to which people focus on others versus the self is deeply rooted in culture^37^. In this respect, one of the most established cultural dimensions is the distinction between collectivism and individualism^38^. While collectivism relates to an interdependent self-concept, individualism is characterized by an independent self-concept^39,40^. This means that in more collectivistic cultures, people tend to focus more strongly on others^41^ in the sense that they are more concerned with the group’s needs, goals, and interests. In contrast, in more individualistic cultures, people are more strongly concerned with their needs, goals, and interests^42^ and thus focus more strongly on the self than on others. While individualistic cultures are predominant in Western countries, such as the USA and several Central-European countries, cultures characterized by higher collectivism are located mainly in East Asia, Africa, and Latin America^43^.

### Present research

Taken together, past research has often treated automatic imitation as a universal phenomenon^1,2^ suggesting that people across the globe share their automatic tendency to imitate others. Although most research on imitation has mainly assessed WEIRD samples, there are a few studies that aimed at testing cultural variations in imitative behavior^44–49^. While some of these studies found cultural variations in imitative behavior, it is important to note that most assessed rather small sample sizes, compared only two countries, often did not compare individualistic with collectivistic nations, and rarely used well-controlled, reliable imitation tasks. Thus, it remains open whether cultural variations in terms of collectivism/individualism modulate people’s automatic tendency to imitate others. Building upon ideomotor theory^15–17^ and the theory of objective self-awareness^29^ proposing that a focus on others (as compared to the self) increases automatic imitation, we predicted that the automatic tendency to imitate others increases the more collectivistic a nation is.

To test this prediction, we carried out two experiments. Experiment 1 assessed automatic imitation in two nations differing in collectivism/individualism (i.e., Vietnam vs. USA). In Experiment 2, we assessed automatic imitation by experimentally controlling for spatial compatibility across 22 nations that differ in collectivism/individualism. Both experiments received ethical approval from the local Ethics Committee of the Faculty of Human Sciences at the University of Cologne, Germany (reference number: OGHF0113). Data, analysis scripts, and stimuli are made accessible on the Open Science Framework (OSF; https://osf.io/uag2k).

## Methods

### Experiment 1

In Experiment 1, we tested whether people from a strongly collectivistic nation imitate more strongly than people from a strongly individualistic nation. Based on the revised Minkov-Hofstede model of culture^50^ we selected Vietnam as a collectivistic nation and the USA as an individualistic nation, because these two nations very strongly differ on the cultural dimension of collectivism/individualism. The experiment, including hypotheses, methods, sample, exclusion criteria, data preprocessing, and analysis, was preregistered at aspredicted.org on September 12th, 2021 (https://aspredicted.org/L6V_6R9).

#### Participants and design

To estimate the sample size required to detect the predicted effect, we conducted an a priori power analysis. We could not base our sample size estimation on previous research, as no studies had been published on the influence of culture on automatic imitation. Thus, we sought to detect a small to medium-sized effect that has still practical relevance^51^. Based on these considerations, for the difference in automatic imitation between nations (i.e., USA vs. Vietnam), we aimed for an effect size of *d* = 0.3, which roughly corresponds to the median effect size in social psychological research^52^. A power calculation with G*Power^53^ revealed that 470 participants are needed to detect such an effect with 90% power and an Alpha-probability of *α* = 0.05. To compensate for preregistered exclusions, we aimed for a sample size of 500 participants (250 in each nation).

To detect effects that generalize beyond student populations, we recruited population-representative samples in terms of age and gender for each nation via the online panel provider Toluna (https://de.toluna.com/). Participants were compensated with Toluna points, with approximately 2000 points awarded for 10 minutes of participation, which could later be redeemed for vouchers (e.g., Amazon, Decathlon, iTunes) or converted into cash at a rate of 4000 points = €1. In total, 506 participants took part in the experiment. In line with our preregistration and previous research using a similar online version of the imitation-inhibition task^54–56^, we discarded participants for whom less than two-thirds of trials (i.e., 40) remained per condition (i.e., congruent and incongruent) after applying trial-based exclusion criteria (*N* = 40) and participants who indicated at the end of the experiment that they did not respond with their right hand (*N* = 67). Nine participants fulfilled both of these criteria.

The final sample consisted of 408 participants (*N*_Vietnam_ = 209; *N*_USA_ = 199), ranging in age from 21 to 65 (*M* = 39.21, SD = 12.65). In total, 207 female and 201 male participants took part in the experiment (for more demographic details, see Table 1). 363 participants reported being right-handed, 33 participants were left-handed, and 12 participants reported being ambidextrous. A power calculation with G*Power^53^ indicated that this sample gave us 85,60% power to detect an effect of *d* = 0.3 with *α* = 0.05.

**Table 1:** Overview of countries

Country	IC	PD	MF	UA	LTO	IN	Culture	Assessed language	N	Age M (SD)	Female	Male
USA	91	40	62	46	26	68	I	English	199	46.51 (11.86)	105	94
Vietnam	20	70	40	30	57	35	C	Vietnamese	209	32.26 (8.92)	102	107

*IC* individualism-collectivism (high values indicate higher ratings for individualism, as compared to collectivism), *PD* power distance, *MF* masculinity-femininity, *UA* uncertainty-avoidance, *LTO* long-term-orientation, *IN* indulgence.

The design consisted of a 2 (Congruency: congruent vs. incongruent trials) x 2 (Nation: USA vs. Vietnam) mixed design, with the first factor within-subjects and the second between-subjects.

#### Procedure

The experiment took place online. Participants first received general information about the experiment and were then informed that their participation was voluntary and that all answers were processed and stored anonymously. Afterwards, they provided informed consent and basic demographic data. Subsequently, they ran through the validated online version^56^ of the imitation-inhibition task^9,10^, which was programmed in JavaScript using the jsPsych library^57^.

When starting the imitation-inhibition task, participants first received basic information about the task and then completed two exercise blocks. In the first exercise block, participants had to respond to randomly appearing number cues (10 trials). That is, they had to press and hold down the “g” key of their computer keyboard with their right index finger and at the same time the “h” key with their right middle finger. When participants pressed both of these keys simultaneously, a fixation cross appeared on the screen for 500 ms, followed by the presentation of either the number “1” or “2”. Participants were instructed to lift their index finger as quickly as possible as soon as the number “1” appeared on the screen and to lift their middle finger as quickly as possible when the number “2” appeared on the screen. The number cue was presented for a maximum duration of 2000 ms or until participants lifted a finger. After each trial, participants received accuracy feedback. To ensure that participants fully understood the procedure, they had to repeat the first exercise block until they reached the threshold of at least eight correct trials.

In the second exercise block (12 trials), as soon as participants pressed and held down the “g” and “h” keys, they saw a picture of another person’s mirrored left hand rotated by 90° counterclockwise for 500 ms. The rotation of the stimuli has been previously used to reduce the impact of spatial compatibility^58,59^.

The other person’s hand wore a blue glove, so participants could not guess the other person’s skin color. To create the impression of a finger movement, the picture of the hand in resting position was followed by a picture of the same hand with either the index or middle finger lifted. At the same time, the number “1” or “2” was presented between the model’s index and middle finger for a maximum of 2000 ms or until participants lifted either their index or middle finger. As in the first exercise block, participants were instructed to respond to the number cues by either lifting the index finger in response to the number “1” or the middle finger in response to the number “2”. Combining hand pictures and number cues resulted in congruent and incongruent trials. In congruent trials, participants had to lift the same finger as the model; in incongruent trials, they had to lift the other finger. Participants received accuracy feedback after each trial and had to repeat the second exercise block until they reached the threshold of at least 8 correct trials.

As soon as participants successfully completed the two exercise blocks, they completed two experimental blocks. The experimental blocks’ structure was similar to the second exercise block. The only differences were that we no longer provided accuracy feedback and increased the number of trials per block to 60. In total, both experimental blocks consisted of 120 trials (60 per congruency condition). Between the experimental blocks, participants could take a self-paced break.

To prepare data for analysis and in line with our preregistration and previous research using a similar version of the imitation-inhibition task^54–56^, we discarded trials faster than 100 ms (0.51%), trials more than 3 SDs below the participant’s mean (1.24%), and trials more than 3 SDs above the participant’s mean (0.06%). For the analysis of the reaction times, we also discarded erroneous trials (7.94%). A Q-Q plot analysis on the congruency effect for Vietnam and USA revealed that the data are normally distributed. Likewise, a Levene test indicated that the variances of the congruency effect in Vietnam and the USA are the same (*p* = 0.244).

At the end of the experiment, participants provided basic demographics and reported with which hand they responded during the task (i.e., with the left hand, the right hand, or both hands). Finally, participants were thanked and dismissed.

Data, analysis scripts, and stimuli are made accessible on the Open Science Framework (OSF; https://osf.io/uag2k).

### Experiment 2

The results of Experiment 1 indicate that there was no difference in automatic imitation between individuals from the USA and individuals from Vietnam. This may suggest that people from individualistic and collectivistic cultures engage to a similar degree in automatic imitation. However, two shortcomings of the applied research design challenge this conclusion.

First, as we assessed the imitation-inhibition task in two countries only, it is unclear whether the detected effects are specific to the USA and Vietnam or whether they can be generalized across more nations that differ in terms of collectivism/individualism. Second, how we assessed automatic imitation may have masked any potential difference in imitative behavior between collectivistic and individualistic nations. Specifically, although we aimed to reduce the influence of spatial compatibility by rotating the image of a model’s left hand counterclockwise by 90°, this procedure may not have completely eliminated the influence of spatial information on participants’ performance. Please note that in Experiment 1, the depicted fingers moved left and right, whereas participants’ fingers moved up and down. This setup could have introduced an orthogonal spatial-compatibility effect, where up-right/down-left advantages emerge due to asymmetric coding of stimulus and response alternatives because upward and rightward movements are typically coded as more salient or positive^60,61^. This nonsocial correspondence effect can confound measurements of automatic imitation, as quicker responses may result not only from imitative tendencies but also from the spatial alignment of stimuli and responses^62^. As a consequence, spatial compatibility could have masked a potential difference in terms of imitative compatibility between individualistic and collectivistic nations.

To address the two shortcomings mentioned above, we conducted Experiment 2, in which we sampled participants from more than two nations and assessed automatic imitation by experimentally controlling for spatial compatibility.

#### Participants

As in Experiment 1, we recruited population-representative samples in terms of age and gender for each nation via the online panel provider Toluna (https://de.toluna.com/). Compensation for participants was the same as in Experiment 1. Nations were selected based on the revised Minkov-Hofstede model of culture^50^. In particular, we selected nations strongly varying on the cultural dimension of collectivism/individualism, ranging from high individualism (USA) to high collectivism (Colombia) while keeping the values on the other cultural dimensions (i.e., power distance, masculinity-femininity, uncertainty-avoidance, long-term orientation, indulgence) between the countries as small as possible. The full list of selected nations, including further information (e.g., demographics, values on the respective cultural dimensions, sample size), can be found in Table 2. Instructions were provided in the respective national languages based on translations made by professional bilingual translators in the respective countries.

**Table 2:** Overview of countries

Country	IC	PD	MF	UA	LTO	IN	Culture	Assessed language	N	Age M (SD)	Female (N)	Male (N)	Diverse (N)
Australia	90	38	61	51	21	71	I	English	190	48.15 (15.52)	97	92	1
Belgium	75	65	54	94	82	57	I	Dutch & French	195	43.96 (14.50)	92	103	0
France	71	68	43	86	63	48	I	French	194	42.78 (13.92)	105	88	1
Germany	67	35	66	65	83	40	I	German	224	45.17 (14.26)	111	111	2
Hungary	80	46	88	82	58	31	I	Hungarian	221	45.79 (13.82)	114	106	1
Italy	76	50	70	75	61	30	I	Italian	206	40.16 (13.72)	101	105	0
Netherlands	80	38	14	53	67	68	I	Dutch	201	42.29 (15.16)	108	92	1
Switzerland	68	34	70	58	74	66	I	German & French	200	44.09 (14.67)	113	87	0
Poland	60	68	64	93	38	29	I	Polish	210	40.50 (14.00)	105	105	0
UK	89	35	66	35	51	69	I	English	209	43.55 (15.21)	114	94	1
USA	91	40	62	46	26	68	I	English	193	46.97 (15.53)	102	90	1
Argentina	46	49	56	86	20	62	C	Spanish	207	41.48 (13.43)	107	99	1
Chile	23	63	28	86	31	68	C	Spanish	196	40.64 (13.96)	107	88	1
Colombia	13	67	64	80	13	83	C	Spanish	203	39.30 (12.69)	109	93	1
Hong Kong	25	68	57	29	61	17	C	English	177	38.84 (12.08)	90	87	0
Indonesia	14	78	46	48	62	38	C	Indonesian	204	35.37 (11.32)	95	109	0
Portugal	27	63	31	99	28	33	C	Portuguese	219	41.20 (14.02)	112	107	0
Singapore	20	74	48	8	72	46	C	English	204	41.41 (12.27)	101	102	1
South Korea	18	60	39	85	100	29	C	Korean	203	41.81 (13.02)	99	104	0
Taiwan	17	48	45	69	93	49	C	Taiwanese	192	36.54 (11.56)	100	92	0
Turkey	37	66	45	85	46	49	C	Turkish	213	37.26 (12.07)	99	113	1
Vietnam	20	70	40	30	57	35	C	Vietnamese	186	33.05 (9.76)	92	94	0

*IC* individualism-collectivism (high values indicate higher ratings for individualism, as compared to collectivism), *PD* power distance, *MF* masculinity-femininity, *UA* uncertainty-avoidance, *LTO* long-term-orientation, *IN* indulgence, *I* individualism, *C* collectivism.

As in Experiment 1, we aimed to recruit 250 participants per nation. In the end, 5573 participants across 22 nations took part in the experiment. Prior to data analysis, we discarded 1126 participants based on the same exclusion criteria we applied in Experiment 1. Specifically, we discarded participants for whom less than two-thirds of trials (i.e., less than 24 trials) remained in one or more of the conditions (*N* = 369) and who reported not having used their right hand during the imitation-inhibition task (*N* = 834). 77 participants fulfilled both of these criteria. After applying these exclusion criteria, 4447 participants (2273 female; 2161 male, 13 diverse) aged 21 to 85 (*M* = 41.42, SD = 14.01) remained in the data set. 4090 (92%) participants reported being born in the country where they currently live. 357 participants reported having moved to the country where they currently live. Those participants who were not born in the country where they currently live, lived there for an average of 25.75 years (SD = 18.75). 3,947 participants reported being right-handed, 352 participants were left-handed, and 148 participants reported being ambidextrous.

To investigate our statistical power to detect the smallest effect size of interest with a sample of 4447 participants, we applied a Monte Carlo simulation–based approach implemented in the R package simr^63^. The smallest effect size of interest was defined as 2.5 ms (as a difference between the experimental conditions or as a difference between −1 SD and +1 SD for analyses involving collectivism). Power was evaluated for the theoretically relevant effects of spatial compatibility, imitative compatibility, their two-way interaction, and their three-way interaction with collectivism. These analyses revealed an estimated power of >99.99% for the main effect of spatial compatibility and imitative compatibility. We observed a 99.60% power for their two-way interaction and a 70.80% power for the three-way interaction with collectivism.

#### Design and procedure

The experiment took place online and the procedure was highly similar to the one implemented in Experiment 1 with one exception. That is, after participants signed an informed consent, we applied the same instructions and exercise blocks as in Experiment 1. In contrast to Experiment 2, however, we changed the stimuli in the experimental blocks to experimentally control for the influence of spatial compatibility by applying the method introduced by Catmur and Heyes^64^. Specifically, we presented images of a left hand from a frontal view in one half of trials and the same hand by vertically mirroring it in the other half of trials (trials were randomly selected). In spatially incompatible trials, the model’s finger movements were located on the other side of space from participants’ finger movements, whereas the model’s finger movements were located on the same side of space as participants’ finger movements in spatially compatible trials. This stimulus setup allowed us to measure automatic imitation as spatially compatible or incompatible within a 2 (Spatial Compatibility: compatible vs. incompatible) × 2 (Imitative Compatibility: compatible vs. incompatible) within-subjects design (for an illustration, see Table 3).

**Table 3:**
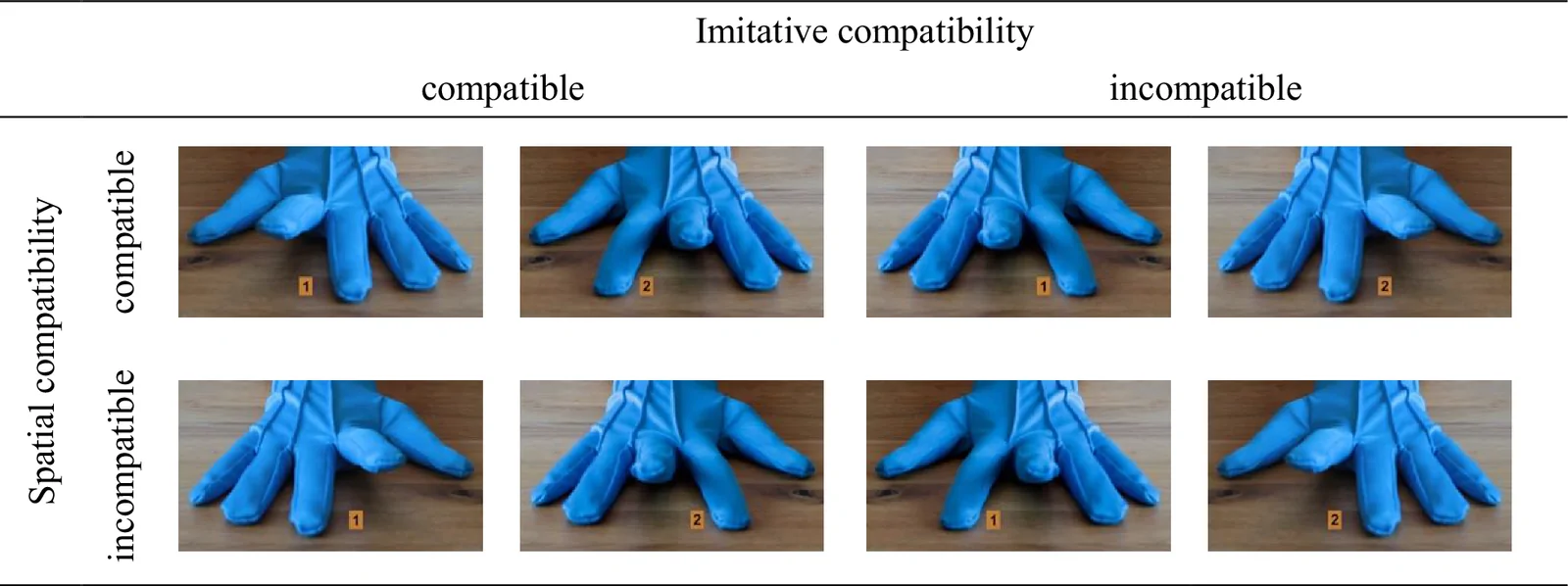
Illustration of the stimuli used in Experiment 2

Participants lifted the right index finger in response to the number “1” and the right middle finger in response to the number “2”.

Participants completed two experimental blocks with 72 trials each (i.e., 144 trials in total). Between the blocks, participants could take a self-paced break. To prepare data for analysis, as in Experiment 1 and previous research using a similar version of the imitation-inhibition task^54–56^, we discarded trials faster than 100 ms (0.21%), trials more than 3 SDs below the participant’s mean (0.03%), and trials more than 3 SDs above the participant’s mean (1.58%). For the analysis of the reaction times, we discarded erroneous trials as well (8.39%). A Q-Q plot on the interaction between spatial and imitative compatibility for collectivistic and individualistic cultures revealed that the data are normally distributed. Likewise, a Levene test indicated that the variances of the interaction between spatial and imitative compatibility are equal (*p* = 0.754).

At the end of the experiment, participants provided basic demographics and indicated with which hand they responded during the task (i.e., with the left hand, the right hand, or both hands). Finally, participants were thanked and dismissed.

Data, analysis scripts, and stimuli are made accessible on the Open Science Framework (OSF; at https://osf.io/uag2k/).

## Results

### Experiment 1

To test our predictions, we carried out, in line with our preregistration, a 2 (Congruency: congruent vs. incongruent trials) x 2 (Nation: USA vs. Vietnam) mixed ANOVA on participant’s reaction times with Nation as a between- and Congruency as a within-subjects factor. We did not consider gender as an additional factor, because male and female participants’ tendency to automatically imitate others is equally strong^11^. The results of the ANOVA yielded a significant main effect of Congruency, *F*(1, 406) = 195.65, *p* < 0.001, *η*_p_² = 0.33, 95% CI [0.26, 0.39], BF_01_ < 0.001, indicating that participants responded faster in congruent trials (*M* = 551.96 ms, SD = 139.62) than in incongruent trials (*M* = 577.62 ms, SD = 137.38). There was also a main effect of Nation, *F*(1, 406) = 26.63, *p* < 0.001, *η*_p_² = 0.06, 95% CI [0.02, 0.11], BF_01_ < 0.001, indicating that participants from Vietnam (*M* = 531.60 ms, SD = 108.71) responded faster than participants from the USA (*M* = 599.65 ms, SD = 154.70). The interaction between Congruency and Nation was not significant, *F* (1, 406) = 1.05, *p* = 0.306, *η*_p_² = 0.003, 95% CI [0.000, 0.021], BF_01_ = 1.360. We report the same analysis of participants’ error rates in the supplemental materials for reasons of transparency.

To further test our predictions, we computed a (non-preregistered) 2 (Congruency: congruent vs. incongruent trials) x 2 (Nation: USA vs. Vietnam) mixed model with reported Bayes factors^65–67^ on participants’ reaction times, including random intercepts for participants. The results yielded a significant effect of Congruency, *B* = 19.99, 95% CI [8.56, 31.42], SE = 5.83, *p* < 0.001, BF_01_ = 0.057, indicating that participants responded faster in congruent trials. There was also a main effect of Nation, *B* = −69.92, 95% CI [−96.01, −43.84], SE = 13.31, *p* < 0.001, BF_01_ < 0.001, indicating that participants from Vietnam (*M* = 531.60 ms, SD = 108.71) responded faster than participants from the USA (*M* = 599.65 ms, SD = 154.70). The interaction between Congruency and Nation was not significant, *B* = 3.75, 95% CI [−3.43, 10.93], SE = 3.66, *p* = 0.306, BF_01_ = 10.68.

### Experiment 2

#### Testing for the presence of automatic imitation

To analyze our data, we firstly investigated the influences of spatial and imitative compatibility on reaction times in a mixed model, including random intercepts for participants (to account for the repeated measurement design) and nations with reported Bayes factors^65–67^. Error rate analyses are reported in the supplement materials for transparency purposes. We did not consider gender as an additional factor, because participants’ tendency to automatically imitate others is equally strong across genders^11^.

Our analysis yielded significant effects for both, spatial compatibility, *B* = −43.66, 95% CI [−44.40, −42.93], *SE* = 0.38, *p* < 0.001, BF_01_ < 0.001, and imitative compatibility, *B* = −6.10, 95% CI [−6.84, −5.35], *SE* = 0.38, *p* < 0.001, BF_01_ < 0.001. In line with previous research^11^, these results indicate that participants responded faster in both types of compatible trials. The interaction between spatial and imitative compatibility was also significant, *B* = −3.75, 95% CI [−4.79, −2.71], SE = 0.53, *p* < 0.001, BF_01_ < 0.001, indicating that overall, the effect of imitative compatibility was larger in spatially compatible than in spatially incompatible trials.

In the next step, we accounted for potential nation-level variability by including random slopes in the model. In this model, the overall effects of spatial compatibility, *B* = −43.73, 95% CI [−45.70, −41.77], SE = 1.00, *p* < 0.001, BF_01_ < 0.001, imitative compatibility, *B* = −6.25, 95% CI [−7.89, −4.60], SE = 0.84, *p* < 0.001, BF_01_ < 0.001 and the interaction between spatial and imitative compatibility, *B* = −3.45, 95% CI [−6.69, −0.21], SE = 1.65, *p* = 0.049, BF_01_ = 0.74 remained significant. Moreover, these analyses revealed that spatial compatibility was associated with faster responses across all 22 nations (see Supplementary Fig. S2).

**Fig. 1:**
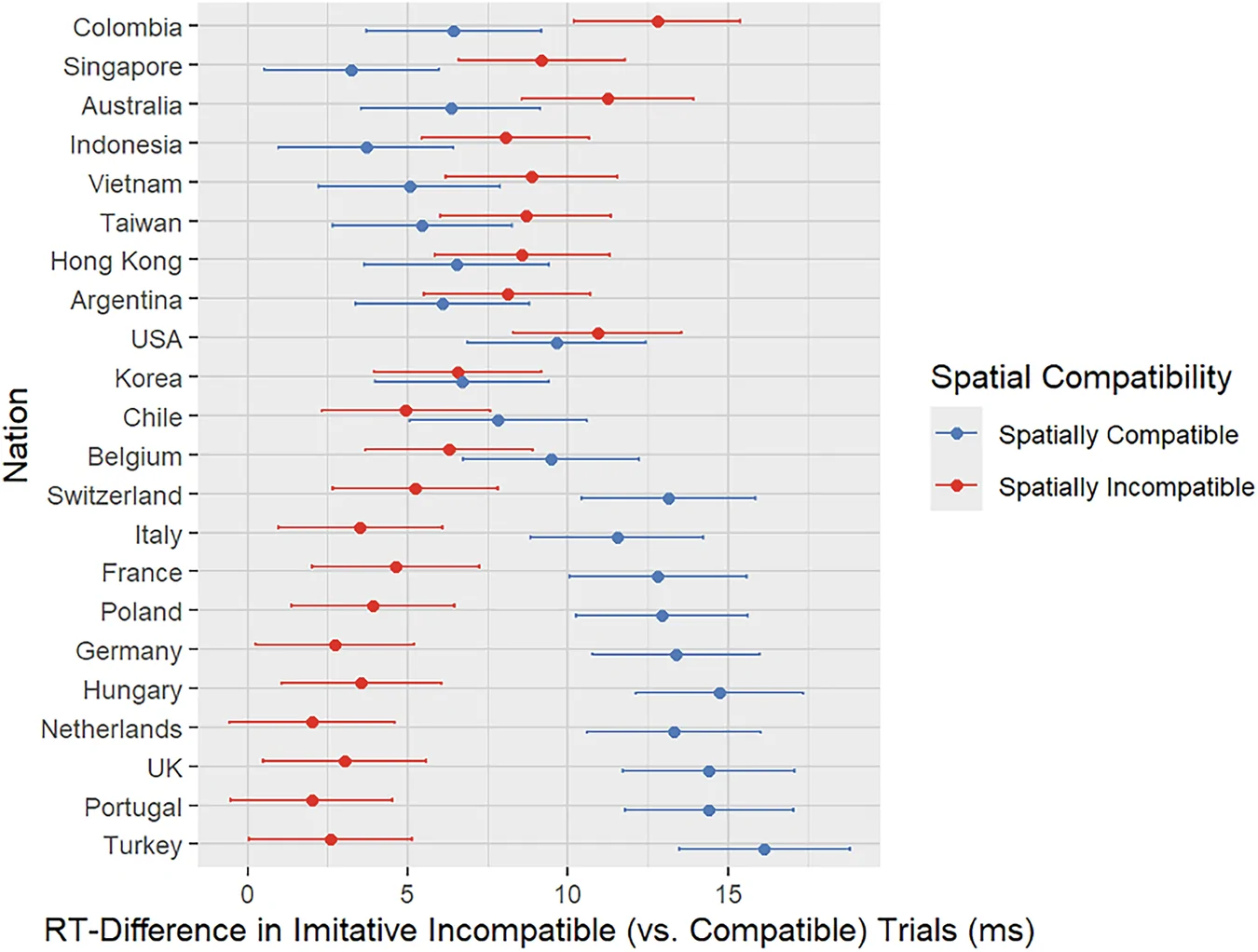
Imitative congruency effects by spatial compatibility and nation. *Note*. Points indicate estimated imitative congruency effects by nation and spatial compatibility. Error bars represent 95% confidence intervals around the estimated effects (*N* = 4447 participants).

We also found strong evidence for universal imitative congruency effects: In spatially compatible trials, we observed effects of imitative compatibility across all nations, indicated by all CIs excluding zero (see Fig. 1). In most nations, this effect also appeared in spatially incompatible trials.

### Relationship with culture

Figure 1 reveals considerable variation regarding the interaction between spatial and imitative compatibility across nations. These findings suggest that spatial compatibility does not universally facilitate the imitative congruency effect. Instead, the interaction between spatial and imitative compatibility seems to depend on the investigated nation. To shed light onto this observation, we investigated whether the degree to which a nation scores on collectivism/individualism can explain the observed national differences. Specifically, we tested whether the observed interaction between imitative and spatial compatibility depends on a nation’s collectivism. To test this, we added national collectivism (note, high values relate to high collectivism and low individualism, whereas low values indicate low collectivism and high individualism) as a continuous factor and its two- and three-way interactions with spatial and imitative compatibility to the mixed model.

This model revealed, based on conventional levels of significance, neither a significant interaction between imitative compatibility and collectivism, *B* = −0.04, 95% CI [−0.10, 0.01], SE = 0.03, *p* = 0.162, BF_01_ = 1.77, nor between spatial compatibility and collectivism, *B* = −0.06, 95% CI [−0.13, 0.01], SE = 0.03, *p* = 0.086, BF_01_ = 1.11. At the same time, the three-way interaction between spatial and imitative compatibility and collectivism, *B* = 0.13, 95% CI [0.02, 0.23], SE = 0.05, *p* = 0.026, BF_01_ = 0.431, reached significance. We probed this three-way interaction by using simple slope analyses. For this, we investigated the two-way interaction between spatial and imitative compatibility at the minimal (0) and maximal (78) levels of collectivism present in our dataset. Spatial compatibility facilitated the imitative congruency effect in highly individualistic cultures, *B* = −8.61, 95% CI [−13.72, −3.50], *SE* = 2.61, *p* = 0.004, BF_01_ = 0.08. In contrast, this interaction was nonsignificant in highly collectivistic cultures, *B* = 1.28, 95% CI [−3.56, 6.13], SE = 2.47, *p* = 0.609, BF_01_ = 3.91.

Using the same procedure, we also investigated the interaction between collectivism and imitative compatibility under both levels of spatial compatibility. In spatially compatible trials, the interaction between imitative compatibility and collectivism/individualism was significant in spatially compatible, *B* = 0.09, 95% CI [0.03, 0.14], SE = 0.03, *p* = 0.011, BF_01_ = 0.21, but not in spatially incompatible trials, *B* = −0.04, 95% CI [−0.10, 0.01], SE = 0.03, *p* = 0.162, BF_01_ = 1.77.

Finally, we investigated the interaction between collectivism and spatial compatibility under both levels of imitative compatibility. This interaction was not significant under imitative compatibility, *B* = 0.07, 95% CI [−0.03, 0.16], SE = 0.05, *p* = 0.208, BF_01_ = 2.10 or under imitative incompatibility, *B* = −0.06, 95% CI [−0.13, 0.01], SE = 0.03, *p* = 0.086, BF_01_ = 1.11.

## General discussion

Previous research suggests that imitation is a global phenomenon in the sense that people generally have the automatic tendency to imitate others. However, since previous research has mainly investigated WEIRD samples and thereby neglected investigating the role of an individual’s cultural background on imitative behavior, it remains open how universal the imitation phenomenon actually is. To fill this gap, we assessed individuals’ tendency to automatically imitate others in 22 nations. Based on ideomotor theory^15–17^ and the theory of objective self-awareness^29^ proposing that a focus on others, as compared to the self, increases automatic imitation, and research demonstrating that in more collectivistic (as compared to individualistic) cultures, people tend to focus more strongly on others than on the self^41,42^, we hypothesized that individuals from more collectivistic nations have a stronger automatic tendency to imitate others than individuals from more individualistic nations. The results of our investigation did not reveal a significant influence of a nation’s level of collectivism/individualism on people’s tendency to automatically imitate others. It is important to note that Bayesian analyses do not reveal strong evidence for this null effect. Thus, the possibility for a modulation by culture remains theoretically plausible. However, as we assessed a large number of diverse nations, a strong modulation by culture seems unlikely. Besides some limitations, these results have several theoretical implications.

Overall, our investigation consistently replicated the basic automatic imitation effect in all investigated nations. Moreover, our findings illustrate that individuals from more collectivistic cultures do not differ from individuals from rather individualistic cultures in terms of how strongly they imitate in general. Indeed, our Bayesian analyses indicate the strongest evidence for the congruency effect and no compelling evidence for a modulation by culture. This supports previous notions that automatic imitation is a universal phenomenon in the sense that people generally have the tendency to automatically imitate other people’s actions^1,2^. At the same time, it limits the generalizability of previous theoretical accounts proposing self-other focus as a key process underlying automatic imitation^15–17,29^ as cultural differences in terms of self-other focus do not seem to modulate imitative behavior in a strong manner.

Experiment 2 also found some qualitative, albeit preliminary, differences in how people imitate. Specifically, we found that spatial compatibility facilitated the imitative congruency effect in highly individualistic cultures (BF_01_ = 0.08), an effect not present in highly collectivistic cultures (BF_01_ = 3.91). This effect may suggest that automatic imitation of people from more individualistic nations is more strongly influenced by spatial information than imitation of people from more collectivistic nations. Different theoretical considerations may explain this finding, while statistical considerations suggest a more careful interpretation. First, it is tempting to assume that, as compared to people from more collectivistic cultures, imitation of people from more individualistic cultures is more strongly influenced by irrelevant external information—that is, spatial information. This interpretation is to some extent in line with research on self-other focus^39,40^ as it supports the notion that collectivism (as compared to individualism) relates to an interdependent self-construal, in the sense that people from more collectivistic nations tend to focus more strongly on others. Our research may indicate that imitation of people from collectivistic (as compared to individualistic) cultures is more robust against non-social influences, as it is less strongly influenced by spatial information. At the same time, our findings may extend the claim that individualism (as compared to collectivism) is characterized by an independent self-concept, as people living in individualistic nations focus more strongly on the self. That is, our findings seem to suggest that people from rather individualistic cultures do not necessarily focus less on other people, but rather relate to cues that are not necessarily social (here, spatial information) when responding to others’ actions.

An alternative interpretation of these results comes from research showing that in compatibility tasks, a higher-level form of congruency exists^68^. In the task we assessed in Experiment 2, higher-level congruency is associated with the finding that it is relatively easier to process information when both forms of compatibility (i.e., imitative and spatial) are congruent rather than incongruent. Our findings may suggest that participants from more individualistic nations have greater difficulty with interference control at a higher level, as this effect is less pronounced in more collectivistic (compared to individualistic) nations. Another alternative interpretation might relate to perspective-taking, as half of the trials used a mirror image of the participants’ hand, while the other half of the trials used an anatomically similar hand rotated by 180 degrees. As meta-analytical evidence indicates that perspective-taking does not relate to automatic imitation^11^, we regard this alternative explanation as rather unlikely though. In sum, the interactions with spatial compatibility may be explained in different manners leading to interesting theoretical implications. At the same time, it is important to note that although both crucial two-way interactions (i.e., spatial compatibility × imitative compatibility in highly individualistic cultures and highly individualistic cultures) reached at least small Bayes Factors, the Bayes Factors for the three-way interaction (i.e., spatial compatibility × imitative compatibility × culture) suggest rather inconclusive evidence for the modulation. Thus, the interactions with culture should be interpreted with caution and considered a preliminary finding, because the effect is not very strong, is only partly supported by Bayesian analyses, and is explained post hoc. Further research is needed to validate this finding and to test the different theoretical explanations explicitly.

Our findings also contribute to the current debate about whether automatic imitation is modulated by social variables^36^. While some social variables, such as animacy (e.g., interacting with a robot or a human)^56^, have been shown to reliably modulate automatic imitation, other research on social modulation of automatic imitation^36,69^ has failed to find consistent patterns (e.g., group membership)^54,70^. In line with the latter findings, our research suggests that social modulation of automatic imitation (at least in a top-down manner) is less strongly pronounced than previously assumed.

### Limitations

Besides these implications, there are some limitations that we need to acknowledge. First, one may critically evaluate the somewhat large number of excluded participants in our research. However, it is important to note that the exclusions were in line with our preregistration, and the number of excluded participants is similarly high in comparison to other research using the same online task to measure automatic imitation^56^. In fact, when we apply the exclusion criteria we and others use, the reliability and effect sizes of the congruency effect observed in online settings match those observed in laboratory settings. We thus do not regard the exclusion rate as problematic.

Second, based on previous literature showing that focusing on others, as compared to the self, increases automatic imitation^31–35^ and research showing that collectivistic (as compared with individualistic) cultures focus more on others than the self^39,40^, we proposed that potential cultural variations in automatic imitation may be due to differences in self-other focus. As we did not measure self-other focus explicitly in the present study, future research should investigate self-other focus more directly to further the understanding of how cultural differences influence imitative behavior.

## Conclusion

Is the automatic tendency to imitate others a global and universal phenomenon? Since previous research on automatic imitation has mainly investigated WEIRD samples, this question has remained open to date. Our investigation across 22 nations varying in collectivism/individualism finds no conclusive evidence for an effect of culture on automatic imitation, hinting to the possibility that automatic imitation is a global, robust phenomenon with little room for strong modulation by culture.

## Supplementary information

Transparent Peer Review file

Supplement Material for: Little Evidence for Cultural Differences in Automatic Imitation

## Data Availability

The datasets generated and analyzed during the current study, including the de-identified behavioral data (reaction times, accuracy, and participant-level variables) and the experimental stimuli, have been deposited in the Open Science Framework (OSF) repository and are publicly available at https://osf.io/uag2k (https://doi.org/10.17605/OSF.IO/UAG2K). The repository serves as the permanent archive for these materials, which are freely accessible without restriction.

## Code availability

The analysis scripts used to analyze the data and produce the figure reported in this study are publicly available in the Open Science Framework (OSF) repository at https://osf.io/uag2k/ (https://doi.org/10.17605/OSF.IO/UAG2K). Statistical analyses were conducted using IBM SPSS Statistics Version 29.0.2.0 (Experiment 1) and R (Experiments 1 and 2) using the packages tidyverse, haven, readxl, lmerTest, psych, sjstats, effectsize, sjPlot, sjmisc, rworldmap, arm, emmeans, effects, and grid. The repository serves as the permanent archive for the analysis scripts, which are freely accessible without restriction.
