# Supplementary material for: Little evidence for cultural differences in automatic imitation
Source: Commun Psychol. 2026 Oct 14;4:139. doi: 10.1038/s44271-026-00517-5 (PMC13661571; doi:10.1038/s44271-026-00517-5)
Supplement: Supplementary file 1 [file 44271_2026_517_MOESM1_ESM.pdf]

nature portfolio

Peer Review File

Little Evidence for Cultural Differences in Automatic Imitation

Corresponding Author: Professor Oliver Genschow

This file contains all reviewer reports in order by version, followed by all author rebuttals in order by version.

Version 0:

Reviewer comments:

Reviewer #1

(Remarks to the Author)
This is an interesting study that investigates cultural influences on automatic imitation in two large-scale online experiments with populations from different cultures. In the first experiment, the authors investigate whether automatic imitation differs between a sample from US and Vietnam. While no effect of the cultural background on automatic imitation was found, the Vietnam sample responded much faster. In the second study, another large-scale study was carried out that investigated samples from several individualistic and collectivist cultures. In this study, spatial and imitative compatibility were dissociated experimentally. While again no effect was found for automatic imitation even when dissociating imitative from spatial compatibility, a three-way interaction was found. I think this is an extremely interesting and well-done study. While I have some remarks regarding the interpretation of the three-way interaction, I think the general pattern of results, in particular the null results are highly relevant and should be published.

1. The conceptualization of spatial compatibility in the current article and in the literature in general is in my opinion problematic. Spatial compatibility is an important aspect of imitation. While it is helpful to dissociate different components in the automatic imitation task, these different components are all part of automatic imitation. Compatibility models like the dimensional overlap model by Kornblum have nicely emphasized this.

2. I think it would be useful to have a figure of the task. In particular, the mapping between spatial and imitative compatibility might be difficult to get for readers outside the research area.

3. There is a strong main effect of nation in Experiment 1 but also in Experiment 2. I wonder whether this might be due to an age difference between the samples. I guess that the mean age of the population in Vietnam is lower than the mean age in the US and this might be reflected in the sampling (if not controlled for).

4. Maybe it would be interesting to compute the interference effect relative to the mean reaction time in each sample. While this is of course a post hoc analysis it might be justified given the unexpected main effect.

5. I think it would be crucial to test the null effect with Bayesian analysis to see how strong the evidence is for the null hypothesis (Experiment 1).

6. The interpretation of the three-way interaction should be treated with caution, as it remains speculative and requires further testing. Furthermore, there are potential alternative interpretations: An alternative interpretation might be that participants from individualistic countries have more problems with interference control on a higher level. There is evidence that in compatibility tasks a higher-level form of congruency (Hedge & Marsh, 1975) exists. In the current task, it might be relatively easier to process information if both forms of compatibility (imitative and spatial) are congruent (compatible-compatible, incompatible-incompatible) compared to a incongruent mappings (compatible-incompatible, incompatible-compatible). While such an effect does not seem to show up in the collectivist sample, it might exist in the individualistic sample. Another alternative interpretation might have to do with perspective-taking. In one experimental condition the hand is a mirror image of participants' hand while in the another condition it is the anatomically same hand rotated by 180 degrees. Perspective taking might apply to different degrees here.

Reviewer #2

(Remarks to the Author)
The study titled ‘Cultural Differences in Automatic Imitation’ investigates an interesting question pertaining to whether automatic imitation is a universal phenomenon or whether it can vary across cultures differing in individualism-collectivism. Study 1 demonstrates that automatic imitation effects are consistent and do not vary between participants belonging to two nations contrasting in individualism-collectivism. Study 2 utilises a task which provides independent measurements of imitative and spatial compatibility effects in a larger sample across 22 nations to demonstrate that the automatic imitation effect replicates in non-WEIRD samples and finds that the imitative compatibility effect was larger in spatially compatible trials for the high extreme of individualism. Overall, the authors suggest that culture might shape how individuals process spatial information whilst engaging in imitative behaviour. Studies of automatic imitation have been largely conducted in WEIRD populations. This study takes an important step in extending the generalizability of automatic imitation effects to diverse populations.

The key conceptual issue I identify in this manuscript is how cultural differences have been measured. Each nation’s sample may be heterogenous in terms of individualism-collectivism due to multiculturalism. There are a few important points which the authors should address to ensure that the presented conclusions are justified by the results:

1. Many countries today are highly multicultural, comprising individuals belonging to different nations who may not have grown up in their country of residence. How did the authors account for individuals who may, for example, have grown up in a “collectivistic” country and have immigrated to an “individualistic” country for work/studies? Since participant-level scores on self-construal were not obtained, it is important to confirm that participants in each nation were broadly homogeneous in terms of culture. Are the country scores (found in supplementary materials) on Hofstede’s indices the mean scores derived from participant-level data? If so, perhaps a Levene’s test can be conducted to determine that all countries have broadly the same variance in individualism-collectivism scores which would support that they are broadly homogeneous.
2. Another limitation which the authors should acknowledge is that individual scores on self-construal were not directly measured to confirm that the three-way interaction between spatial compatibility by imitative compatibility by collectivism was indeed driven by differences in one’s self-construal, as the authors suggest.
3. This study finds that spatial and imitative compatibility interacted with collectivism when considering the extreme points of this factor. While this is interesting, it should be considered as a preliminary finding, at best, considering that a weak effect was only found at the extreme points of individualism-collectivism, and considering that individual differences in self-construal were measured indirectly. The conclusions and claims presented in the study, should therefore, be toned down to reflect the preliminary nature of these findings.
4. The introduction section should present a more balanced review of the literature concerning the putative modulating effect of self-focus on automatic imitation. The evidence suggesting that self-focus (and other external social factors) may modulate automatic imitation is not very convincing, something that the authors have acknowledged in their previous reviews.

Minor point: There seems to be a typo on page 12 where Colombia is misspelled as ‘Columbia’.

Overall, this is an interesting piece of work which extends previously reported findings related to automatic imitation to diverse populations beyond WEIRD samples; something which is currently lacking in the field.

Dr. Divyush Khemka

Reviewer #3

(Remarks to the Author)
I read the manuscript entitled “Cultural Differences in Automatic Imitation” by Genschow and colleagues with interest. There are many commendable aspects of the manuscript and the research. The write-up is a highly effective, concise presentation of the work. Below, I raise some points for consideration and attempt to respond to the questions posed by the journal in its instructions for reviewers.
1. In my view, the authors’ effort represents a significant advance in our understanding of automatic imitation by exploring the previously neglected possibility of cultural influences on imitation. Experiment 1 presents an initial test and Experiment 2 presents a deeper exploration. By including age- and gender-representative samples from 22 cultures and including spatial compatibility in the design, the authors reveal hitherto unknown patterns of imitation that seems highly likely to inspire further research. In sum, I consider the present findings original and strongly worthy of attention in this area of research.
2. A strong aspect of the research is that Experiment 1 was preregistered and this preregistration matches the manuscript’s report of the procedures. Experiment 2 does not seem to be preregistered. However, it closely follows Experiment 1 (e.g., in data exclusion criteria). Thus, the lack of preregistration in Experiment 2 did not have any impact on my evaluation of the transparency and robustness of the effort.
3. I did not encounter “any special ethical concerns arising from the use of animals or human subjects” in the present research. A recently published effort validates the use of the online version of the imitation task, reassuring the reader regarding analysis of reaction time data collected over the internet. There were at least two other sources cited to support the reliability and validity of the main task used in these experiments. All of this pointed to strong and meticulous methods. I could provide one comment about the samples: It is a strong point that the samples are age- and gender-representative. However, especially given my lack of familiarity with the recruitment service (Toluna), perhaps some other information about sample characteristics (beyond Table 2 in the Supplementary Materials) could be provided to ease concerns about ecological fallacy. For instance, samples from certain cultures that are enrolled in the recruitment service (or more broadly with consistent internet access) may be self-selected (e.g., especially individualistic compared to the rest of their culture).

4. On line 243, there is a minus sign for the F-value, which is impossible. It may be good to run the manuscript through statcheck.
5. Were the exercise blocks removed in Experiment 2? Is there any downside to this or how is it justified?
6. The authors could specify the analytic strategy for Experiment 2. It appears to be a 3-level multilevel linear mixed model. Could the chosen target of n = 250 per culture be better justified? I admit that power analysis for MLM designs could get complicated but my understanding is that this study is likely well-powered. The amount of power (e.g., for detecting a small or medium) effect could be computed and presented to give the reader this reassurance.
7. Starting on line 442, the authors bring “visual scene detection” research to help explain the current findings. Many social psychology findings have received scrutiny in recent times and frequently the original effect is irreplicable or the effect size is smaller in high-quality replications. It might be wise to check if this is the case for these studies. For instance, Grossman and Jowhari (2018) and Drouin and Davidson (2015) both report diverging findings in replications of Kühnen and Oyserman (2002).
8. The authors make the point that imitation research has largely relied on WEIRD cultures, which seems to be true. However, perhaps they could provide some more information on what non-WEIRD research (and cross-cultural comparisons, as well as studies focusing on self-construal) has been conducted and what was found. For instance, could the studies finding cultural differences in children’s imitative behaviors (e.g., Berl & Hewlett, 2015; Clegg & Legare, 2016; Corriveau et al., 2013, 2017; DiYanni et al., 2015) be relevant here in terms of giving a broader context to the current effort?
9. The effort generally provides strong evidence for its conclusions. There is a clear pattern of differences in imitative behavior that involves culture. At the same time, the conclusions partly rest on the absence of evidence (e.g., for an interaction involving culture). Because this is a tricky issue within the frequentist framework, its further discussion could be considered.
On a related note, it could always be that power was insufficient to capture the interaction in Experiment 1 (or the difference in individualism-collectivism in the USA vs Vietnam contrast not strong enough). This is probably less important now given the presence of Experiment 2 but in any case, the authors could describe their sample size estimation strategy more clearly by stating precisely what the target effect was (e.g., is it for detecting an interaction effect in a 2*2 mixed ANOVA). I do not have G*Power handy because I’m on Linux but I was unable to reproduce the estimated sample size. It’s likely because what I’m using requires specifying many assumptions that G*Power does not ask for. However, it also seems to me that the estimated sample size was not for detecting an interaction effect. There is also the additional issue that while the targeted sample size was 250 per culture in Experiment 1, the realized sample size fell short. Thus, what was the realized power?

In conclusion, I find the present research to be interesting, well-executed, and effectively presented. Attention to some remaining issues should make this manuscript worthy of publication in a high-quality outlet. I wish the authors good luck in their continued effort.
Adil Saribay
-I always sign my reviews

Version 1:

Reviewer comments:

Reviewer #1

(Remarks to the Author)
The authors have completely addressed my comments.

Reviewer #2

(Remarks to the Author)
The revised manuscript titled ‘Little evidence for cultural differences in automatic imitation’ has addressed all of my comments made during the initial review. I have no further comments. Thank you to all the authors for their work on the initial submission and subsequent amendments.
Dr. Divyush Khemka

Open Access This Peer Review File is licensed under a Creative Commons Attribution 4.0 International License, which permits use, sharing, adaptation, distribution and reproduction in any medium or format, as long as you give appropriate credit to the original author(s) and the source, provide a link to the Creative Commons license, and indicate if changes were made.
In cases where reviewers are anonymous, credit should be given to 'Anonymous Referee' and the source.
The images or other third party material in this Peer Review File are included in the article's Creative Commons license, unless indicated otherwise in a credit line to the material. If material is not included in the article's Creative Commons license and your intended use is not permitted by statutory regulation or exceeds the permitted use, you will need to obtain permission directly from the copyright holder.
To view a copy of this license, visit https://creativecommons.org/licenses/by/4.0/

Yafeng Pan, PhD
Editorial Board Member
Communications Psychology

Revision: Cultural Differences in Automatic Imitation

Dear Prof. Pan,

We would like to thank you and the Reviewers for the very helpful and constructive comments we have received in response to our submission. We have now carefully addressed all the comments.

In the following pages, we have detailed how we have addressed each comment raised by you and the Reviewers. To indicate how we responded to the comments, we cite from the editorial letter and the reviews in italics and subsequently describe in detail how we dealt with the comments marked by →.

In sum, we believe that we successfully addressed all the issues that were raised by you and the Reviewers. Most importantly, we believe that the manuscript greatly profited from the changes we have made, and we hope that you share this impression. Please, let us know if there are any further issues that you would like us to address.

We are looking forward to hearing from you.

Yours sincerely,
Oliver Genschow

Editorial Letter

Dear Professor Genschow,

Thank you for your patience during the peer-review process. Your manuscript titled "Cultural Differences in Automatic Imitation" has now been seen by 3 reviewers, and I include their comments at the end of this message. They find your work of interest but raised some important points. We are interested in the possibility of publishing your study in Communications Psychology, but would like to consider your responses to these concerns and assess a revised manuscript before we make a final decision on publication.

We therefore invite you to revise and resubmit your manuscript, along with a point-by-point response to the reviewers. Please highlight all changes in the manuscript text file.

→ We would like to thank you and the Reviewers for this overall positive evaluation of our manuscript.

Editorially, we consider that the authors should particularly address: (1) the interpretation of the three-way interaction, (2) the assessment of cultural differences (i.e., how they were measured, as raised by R2), and (3) a clearer introduction/discussion of existing non-WEIRD research and its findings.

→ We incorporated all suggestions of the Reviewers. For details, please see our response to the specific comments below.

Please ensure you follow our statistical guidelines when reporting statistics (https://www.nature.com/commspsychol/submit/submission-guidelines#statistical-guidelines). Please note in particular our requirements for the reporting and interpretation of null-results. Non-significant findings derived from null-hypotheses significance tests should be reported in full, but may not be interpreted. Where you interpret null results, this interpretation must be based on Bayes Factors or equivalence tests. Please add appropriate stats and include a sensitivity power analysis.

→ In line with these requests, we report non-significant findings derived from null-hypotheses significance tests in full, but interpret null results only based on Bayes Factors. Also, we included sensitivity power analyses.

As you revise the manuscript in response to these issues, please also implement all requests in the attached Mandatory Revision Requests document. All requirements listed in this document need to be fully met, or the work will be returned to you for further revisions without peer review. This workflow is in place to increase the likelihood that the paper will be accepted for publication. It reduces the number of rounds of revision (and review) and ensures that the reviewers vet a version of the article that is compliant with journal policies. If you have any questions regarding the required revisions, please contact the journal prior to resubmission to avoid a negative outcome.

→ We implemented all requests as suggested in the Mandatory Revision Requests document

We are committed to providing a fair and constructive peer-review process. Please

don't hesitate to contact us if you wish to discuss the revision in more detail.

Please submit the following items:

- *Revised manuscript*
- *Point-by-point response to the referees' comments*
- *Mandatory Revision Requests Table (attached).*
- *Cover letter (as a separate document)*

via this link: https://mts-commspsychol.nature.com/cgi-bin/main.plex?el=A2DU6rZ7A3FLR1I7A9ftdzhKaNp4sUMGuwnkx0VWgZ .

*** This url links to your confidential home page and associated information about manuscripts you may have submitted or are reviewing for us. If you wish to forward this email to co-authors, please delete the link to your homepage first ***

We hope to receive your revised paper within 8 weeks; please let us know if you aren't able to submit it within this time so that we can discuss how best to proceed. If we don't hear from you, and the revision process takes significantly longer, we may close your file. In this event, we will still be happy to reconsider your paper at a later date, provided it still presents a significant contribution to the literature at that stage.

We look forward to seeing the revised manuscript and thank you for the opportunity to review your work.

Best regards,
Yafeng Pan

Reviewer #1 (Remarks to the Author):

This is an interesting study that investigates cultural influences on automatic imitation in two large-scale online experiments with populations from different cultures. In the first experiment, the authors investigate whether automatic imitation differs between a sample from US and Vietnam. While no effect of the cultural background on automatic imitation was found, the Vietnam sample responded much faster. In the second study, another large-scale study was carried out that investigated samples from several individualistic and collectivist cultures. In this study, spatial and imitative compatibility were dissociated experimentally. While again no effect was found for automatic imitation even when dissociating imitative from spatial compatibility, a three-way interaction was found. I think this is an extremely interesting and well-done study. While I have some remarks regarding the interpretation of the three-way interaction, I think the general pattern of results, in particular the null results are highly relevant and should be published.
→ Thank you very much for the positive evaluation of our research.

1. The conceptualization of spatial compatibility in the current article and in the literature in general is in my opinion problematic. Spatial compatibility is an important aspect of imitation. While it is helpful to dissociate different components in the automatic imitation task, these different components are all part of automatic imitation. Compatibility models like the dimensional overlap model by Kornblum have nicely emphasized this.
→ We very much like this point and now more explicitly argue that spatial compatibility can be considered as part of imitation, referring to the proposed model by Kornblum. On p. 4 we now write:

> "By explaining how stimulus and response dimensions in typical S-R tasks overlap, Kornblum et al.[1] argue that responses speed up, the more dimensions of a response overlap with the presented stimulus. In line with this view, imitative responses are faster when the movements are spatially aligned[2], suggesting that the spatial dimension of a movement is an important aspect of automatic imitation."

2. I think it would be useful to have a figure of the task. In particular, the mapping between spatial and imitative compatibility might be difficult to get for readers outside the research area.
→ As suggested, we added a figure that displays the mapping between spatial and imitative compatibility. We added the figure on p. 15.

3. There is a strong main effect of nation in Experiment 1 but also in Experiment 2. I wonder whether this might be due to an age difference between the samples. I guess that the mean age of the population in Vietnam is lower than the mean age in the US and this might be reflected in the sampling (if not controlled for).
→ Indeed, as the Reviewer pointed out, samples were representative for the respective countries in terms of age and gender. Thus, in line with the Reviewer's recommendation, we added analyses controlling for age. It is important to note though that these additional analyses do not change the pattern of results. We report these analyses in the supplementary material.

4. Maybe it would be interesting to compute the interference effect relative to the mean reaction time in each sample. While this is of course a post hoc analysis it might be justified given the unexpected main effect.
→ We agree that differences in baseline reaction time across samples are an important consideration when interpreting the interference effect. Importantly, all reported analyses in Experiment 2 were conducted using multilevel models that include random intercepts for country, which account for differences in mean reaction time between samples, so the different baseline levels in reaction time are already controlled for. For consistency reasons, we now report the same analysis for Experiment 1 also.

5. I think it would be crucial to test the null effect with Bayesian analysis to see how strong the evidence is for the null hypothesis (Experiment 1).
→ We appreciate the Reviewer's recommendation to report Bayes Factors. Going even further than this recommendation, we now report Bayes Factors for all analyses.

6*. The interpretation of the three-way interaction should be treated with caution, as it remains speculative and requires further testing. Furthermore, there are potential alternative interpretations: An alternative interpretation might be that participants from individualistic countries have more problems with interference control on a higher level. There is evidence that in compatibility tasks a higher-level form of congruency (Hedge & Marsh, 1975) exists. In the current task, it might be relatively easier to process information if both forms of compatibility (imitative and spatial) are congruent (compatible-compatible, incompatible-incompatible) compared to a incongruent mappings (compatible-incompatible, incompatible-compatible). While such an effect does not seem to show up in the collectivist sample, it might exist in the individualistic*

sample. Another alternative interpretation might have to do with perspective-taking. In one experimental condition the hand is a mirror image of participants' hand while in the another condition it is the anatomically same hand rotated by 180 degrees. Perspective taking might apply to different degrees here.

→ We greatly appreciated these alternative explanations and agree with the Reviewer that the three-way interaction involving spatial compatibility should be treated with caution. Thus, within the whole manuscript we toned down (see also our response to the comments of Reviewer 2) the interpretation of the three-way interaction and now mainly label it as preliminary evidence. Moreover, in the General Discussion on p. 21 ff, we added:

> "An alternative interpretation of the results comes from research showing that in compatibility tasks, a higher-level form of congruency exists[3]. In the task we assessed in Experiment 2, higher-level congruency is associated with the finding that it is relatively easier to process information when both forms of compatibility (i.e., imitative and spatial) are congruent rather than incongruent. Our findings suggest that participants from more individualistic nations have greater difficulty with interference control at a higher level, as this effect is less pronounced in more collectivistic (compared to individualistic) nations. Another alternative interpretation might relate to perspective-taking, as half of the trials used a mirror image of the participants' hand, while the other half of the trials used an anatomically similar hand rotated by 180 degrees. As meta-analytic evidence indicates that perspective-taking does not relate to automatic imitation[2], we regard this alternative explanation as rather unlikely though. In sum, the interaction with spatial compatibility may be explained in different manners leading to interesting theoretical implications. At the same time, the detected three-way interaction should be interpreted with caution and considered a preliminary finding, because the effect was not very strong, only weakly supported by Bayesian analyses, and explained post hoc. Further research is needed to validate this finding and to test the different theoretical explanations explicitly."

Reviewer #2 (Remarks to the Author):

The study titled 'Cultural Differences in Automatic Imitation' investigates an interesting question pertaining to whether automatic imitation is a universal phenomenon or whether it can vary across cultures differing in individualism-collectivism. Study 1 demonstrates that automatic imitation effects are consistent and do not vary between participants belonging to two nations contrasting in individualism-collectivism. Study 2 utilises a task which provides independent measurements of imitative and spatial compatibility effects in a larger sample across 22 nations to demonstrate that the automatic imitation effect replicates in non-WEIRD samples and finds that the imitative compatibility effect was larger in spatially compatible trials for the high extreme of individualism. Overall, the authors suggest that culture might shape how individuals process spatial information whilst engaging in imitative behaviour. Studies of automatic imitation have been largely conducted in WEIRD populations. This study takes an important step in extending the generalizability of automatic imitation effects to diverse populations.

The key conceptual issue I identify in this manuscript is how cultural differences have been measured. Each nation's sample may be heterogenous in terms of individualism-collectivism due to multiculturalism. There are a few important points

which the authors should address to ensure that the presented conclusions are justified by the results:

1. Many countries today are highly multicultural, comprising individuals belonging to different nations who may not have grown up in their country of residence. How did the authors account for individuals who may, for example, have grown up in a "collectivistic" country and have immigrated to an "individualistic" country for work/studies? Since participant-level scores on self-construal were not obtained, it is important to confirm that participants in each nation were broadly homogeneous in terms of culture. Are the country scores (found in supplementary materials) on Hofstede's indices the mean scores derived from participant-level data? If so, perhaps a Levene's test can be conducted to determine that all countries have broadly the same variance in individualism-collectivism scores which would support that they are broadly homogeneous.

→ Thank you very much for these important questions. With regard to the first question, we asked participants where they were born. Analyzing these data reveals that the vast majority of participants was born in the country where they are currently living. So, we do not regard multiculturalism as an issue in our study. On p. 12, we report these statistics in more detail:

> "4,090 (92%) participants reported being born in the country where they currently live. 357 participants reported having moved to the country where they currently live. Those participants who were not born in the country where they currently live, lived there for an average of 25.75 years (SD = 18.75)."

Regarding the second question, the country scores reported in the supplementary materials for Hofstede's indices are not the mean scores derived from participant-level data. We took these data provided on the Hofstede website (2021).

2. Another limitation which the authors should acknowledge is that individual scores on self-construal were not directly measured to confirm that the three-way interaction between spatial compatibility by imitative compatibility by collectivism was indeed driven by differences in one's self-construal, as the authors suggest.

→ In line with the Reviewer's recommendation, we now explicitly mention this limitation in the General Discussion. On p. 23 we now write:

> "Second, based on previous literature showing that focusing on others, as compared to the self, increases automatic imitation[4-8] and research showing that collectivistic (as compared with individualistic) cultures focus more on others than the self[9,10], we proposed that potential cultural variations in automatic imitation may be due to differences in self-other focus. As we did not measure self-other focus explicitly in the present study, future research should investigate self-other focus more directly to further the understanding of how cultural differences influence imitative behavior."

3. This study finds that spatial and imitative compatibility interacted with collectivism when considering the extreme points of this factor. While this is interesting, it should be considered as a preliminary finding, at best, considering that a weak effect was only found at the extreme points of individualism-collectivism, and considering that individual differences in self-construal were measured indirectly. The conclusions and claims presented in the study, should therefore, be toned down to reflect the preliminary nature of these findings.

→ As suggested by the Reviewer, we now label these findings as preliminary evidence and interpret the results more cautiously. We do this at different parts of the manuscript.

In the Abstract, we now write:

> “There was preliminary evidence for the influence of how non-social cues shape imitation, as automatic imitation of participants from individualistic nations was more strongly influenced by spatial information, whereas imitation of individuals from collectivistic nations was more robust against these non-social influences.”

In the General Discussion, we wrote:

> “Experiment 2 also found some qualitative, albeit small, differences in how people imitate. (…) At the same time, the detected three-way interaction should be interpreted with caution and considered a preliminary finding, because the effect is not very strong, is only weakly supported by Bayesian analyses, and is explained post hoc. Moreover, as we selected nations that broadly differ in regard to collectivism/individualism, the small effect we found might even been an overestimation of the real interaction. Thus, further research is needed to validate this finding and to test the different theoretical explanations explicitly.”

4. The introduction section should present a more balanced review of the literature concerning the putative modulating effect of self-focus on automatic imitation. The evidence suggesting that self-focus (and other external social factors) may modulate automatic imitation is not very convincing, something that the authors have acknowledged in their previous reviews.

→ In line with the Reviewer’s suggestion, we now present a more balanced review of the literature on the influence of self-other focus on imitative behavior. In particular, on p. 5 we now write:

> “It is important to note that these findings should be considered as preliminary evidence for the idea that higher-order processes involved in self-other focus influence imitative behavior, as the literature produced mixed results[11].”

Minor point: There seems to be a typo on page 12 where Colombia is misspelled as ‘Columbia’.

→ Thanks for spotting the error. We corrected it.

Overall, this is an interesting piece of work which extends previously reported findings related to automatic imitation to diverse populations beyond WEIRD samples; something which is currently lacking in the field.
→ Thank you very much for this overall positive evaluation!

Dr. Divyush Khemka

Reviewer #3 (Remarks to the Author):

I read the manuscript entitled "Cultural Differences in Automatic Imitation" by Genschow and colleagues with interest. There are many commendable aspects of the manuscript and the research. The write-up is a highly effective, concise presentation of the work. Below, I raise some points for consideration and attempt to respond to the questions posed by the journal in its instructions for reviewers.

1. In my view, the authors' effort represents a significant advance in our understanding of automatic imitation by exploring the previously neglected possibility of cultural influences on imitation. Experiment 1 presents an initial test and Experiment 2 presents a deeper exploration. By including age- and gender-representative samples from 22 cultures and including spatial compatibility in the design, the authors reveal hitherto unknown patterns of imitation that seems highly likely to inspire further research. In sum, I consider the present findings original and strongly worthy of attention in this area of research.
→ Thank you very much for this positive evaluation.

2. A strong aspect of the research is that Experiment 1 was preregistered and this preregistration matches the manuscript's report of the procedures. Experiment 2 does not seem to be preregistered. However, it closely follows Experiment 1 (e.g., in data exclusion criteria). Thus, the lack of preregistration in Experiment 2 did not have any impact on my evaluation of the transparency and robustness of the effort.
→ We agree with this assessment.

3. I did not encounter "any special ethical concerns arising from the use of animals or human subjects" in the present research. A recently published effort validates the use of the online version of the imitation task, reassuring the reader regarding analysis of reaction time data collected over the internet. There were at least two other sources cited to support the reliability and validity of the main task used in these experiments. All of this pointed to strong and meticulous methods. I could provide one comment about the samples: It is a strong point that the samples are age- and gender-representative. However, especially given my lack of familiarity with the recruitment service (Toluna), perhaps some other information about sample characteristics (beyond Table 2 in the Supplementary Materials) could be provided to ease concerns about ecological fallacy. For instance, samples from certain cultures that are enrolled in the recruitment service (or more broadly with consistent internet access) may be self-selected (e.g., especially individualistic compared to the rest of their culture).
→ In response to the Reviewer's comment, we checked whether the cultures we assessed differ in terms of internet access and usage at the time we collected the data (i.e., year 2023). When analyzing how many people in each country had internet access and used it regularly, we did not find any meaningful differences across

cultures. We thus do not regard self-selection based on internet access as very likely. We report the analysis in the supplement materials on p. 2-3:

> "We checked whether there is a difference between cultures in terms of internet usage by analyzing internet usage data from the year 2023[1]—the year in which we conducted Experiment 2. There was no statistically meaningful difference in the percent of the population using the internet between collectivistic (*M* = 87.00 %; *SD* = 8.98) and individualistic cultures (*M* = 92.55 %; *SD* = 4.30), *t*(20) = 1.85, *p* = .079, *d* = .79, 95% CI [-0.71, 11.81]. A Bayes Factor computed in JASP with the default priors (i.e., Cauchy prior width) of 0.707 to test for evidence that there is a difference between the cultures yielded BF_{10} = 1.241 indicating that there is not much evidence for the idea that the two cultures differ in terms of internet usage."

4. On line 243, there is a minus sign for the F-value, which is impossible. It may be good to run the manuscript through statcheck.
→ Thank you very much for spotting this issue. We corrected the error and double-checked all reported values via statcheck.

5. Were the exercise blocks removed in Experiment 2? Is there any downside to this or how is it justified?
→ Thank you very much for raising this question. It made us realize that we did not report the method clearly enough. In fact, we included the same exercise blocks in Experiment 2 as in Experiment 1. We now better explain this in the manuscript on p. 14 when writing:

> "(…) we applied the same instructions and exercise blocks as in Experiment 1."

6. The authors could specify the analytic strategy for Experiment 2. It appears to be a 3-level multilevel linear mixed model. Could the chosen target of n = 250 per culture be better justified? I admit that power analysis for MLM designs could get complicated but my understanding is that this study is likely well-powered. The amount of power (e.g., for detecting a small or medium) effect could be computed and presented to give the reader this reassurance.
→ In line with the Reviewer's suggestion, we now report a power analysis. On p. 13 we now write:

> "To investigate our statistical power to detect the smallest effect size of interest with a sample of 4,447 participants, we applied a Monte Carlo simulation–based approach implemented in the R package simr[13]. The smallest effect size of interest was defined as 2.5 ms (as a difference between the experimental conditions or as a difference between −1 SD and +1 SD for analyses involving collectivism). Power was evaluated for the theoretically relevant effects of spatial compatibility, imitative compatibility, their two-way interaction, and their three-way interaction with collectivism. These analyses revealed an estimated power of > 99.99% for the main effect of spatial compatibility and imitative compatibility. We observed a 99.60% power for their two-way interaction and a 70.80% power for the three-way interaction with collectivism."

7. Starting on line 442, the authors bring "visual scene detection" research to help explain the current findings. Many social psychology findings have received scrutiny in recent times and frequently the original effect is irreplicable or the effect size is

smaller in high-quality replications. It might be wise to check if this is the case for these studies. For instance, Grossman and Jowhari (2018) and Drouin and Davidson (2015) both report diverging findings in replications of Kühnen and Oyserman (2002).
→ We would like to thank the Reviewer for hinting us to this highly relevant literature. After reading it, we decided to delete the paragraph in question because the cited literature seems difficult to replicate.

8. The authors make the point that imitation research has largely relied on WEIRD cultures, which seems to be true. However, perhaps they could provide some more information on what non-WEIRD research (and cross-cultural comparisons, as well as studies focusing on self-construal) has been conducted and what was found. For instance, could the studies finding cultural differences in children's imitative behaviors (e.g., Berl & Hewlett, 2015; Clegg & Legare, 2016; Corriveau et al., 2013, 2017; DiYanni et al., 2015) be relevant here in terms of giving a broader context to the current effort?
→ We thank the Reviewer for hinting us to this very interesting literature. We included these references and added references from an additional literature review. On p. 6 we now write:

> "Although most research on imitation has mainly assessed WEIRD samples, there are a few studies that aimed at testing cultural variations in imitative behavior[14-19]. While some of these studies found cultural variations in imitative behavior, it is important to note that most assessed rather small sample sizes, compared only two countries, often did not compare individualistic with collectivistic nations, and rarely used well-controlled, reliable imitation tasks. Thus, it remains open whether cultural variations in terms of collectivism/individualism modulate people's automatic tendency to imitate others."

9. The effort generally provides strong evidence for its conclusions. There is a clear pattern of differences in imitative behavior that involves culture. At the same time, the conclusions partly rest on the absence of evidence (e.g., for an interaction involving culture). Because this is a tricky issue within the frequentist framework, its further discussion could be considered.
→ Thank you for this comment. To better quantify the evidence for H0 vs. H1, we have now added Bayesian statistics to all our analyses.

*On a related note, it could always be that power was insufficient to capture the interaction in Experiment 1 (or the difference in individualism-collectivism in the USA vs Vietnam contrast not strong enough). This is probably less important now given the presence of Experiment 2 but in any case, the authors could describe their sample size estimation strategy more clearly by stating precisely what the target effect was (e.g., is it for detecting an interaction effect in a 2*2 mixed ANOVA). I do not have G*Power handy because I'm on Linux but I was unable to reproduce the estimated sample size. It's likely because what I'm using requires specifying many assumptions that G*Power does not ask for. However, it also seems to me that the estimated sample size was not for detecting an interaction effect. There is also the additional issue that while the targeted sample size was 250 per culture in Experiment 1, the realized sample size fell short. Thus, what was the realized power?*

→ In line with the Reviewer's suggestion, we now report achieved power for both experiments. On p. 7, we now better describe the effect size estimate and power calculations for Experiment 1:

> "(…) we sought to detect a small to medium-sized effect that has still practical relevance[20]. Based on these considerations, for the difference in automatic imitation between nations (i.e., USA vs. Vietnam), we aimed for an effect size of *d* = .3, which roughly corresponds to the median effect size in social psychological research[21]. A power calculation with G*Power[22] revealed that 470 participants are needed to detect such an effect with 80 % power and an Alpha-probability of *α* = .05."

On 8, we then report how much power we achieved after participant exclusions:

> "A power calculation with G*Power[22] indicated that this sample gave us 85,60 % power to detect an effect of *d* = .3 with *α* = .05."

For Experiment 2, we added further power considerations. In particular, on p. 12 we now write:

> "As in Experiment 1, we aimed to recruit 250 participants per nation."

In addition, on p. 13-14 we now write:

> "To investigate our statistical power to detect the smallest effect size of interest with a sample of 4,447 participants, we applied a Monte Carlo simulation–based approach implemented in the R package simr[13]. The smallest effect size of interest was defined as 2.5 ms (as a difference between the experimental conditions or as a difference between −1 SD and +1 SD for analyses involving collectivism). Power was evaluated for the theoretically relevant effects of spatial compatibility, imitative compatibility, their two-way interaction, and their three-way interaction with collectivism. These analyses revealed an estimated power of > 99.99% for the main effect of spatial compatibility and imitative compatibility. We observed a 99.60% power for their two-way interaction and a 70.80% power for the three-way interaction with collectivism."

In conclusion, I find the present research to be interesting, well-executed, and effectively presented. Attention to some remaining issues should make this manuscript worthy of publication in a high-quality outlet. I wish the authors good luck in their continued effort.
Adil Saribay
-I always sign my reviews
→ Thank you very much for your insightful and constructive evaluation!

References

1 Kornblum, S., Hasbroucq, T. & Osman, A. Dimensional overlap: cognitive basis for stimulus-response compatibility--a model and taxonomy. *Psychological Review* **97**, 253-270 (1990).

2 Cracco, E. *et al.* Automatic imitation: A meta-analysis. *Psychological Bulletin* **144**, 453-500 (2018). https://doi.org:https://doi.org/10.1037/bul0000143
3 Hedge, A. & Marsh, N. The effect of irrelevant spatial correspondences on two-choice response-time. *Acta psychologica* **39**, 427-439 (1975).
4 Cracco, E., Clauwaert, A., Van den Broeck, Y., Van Damme, S. & Brass, M. Motor simulation is disturbed when experiencing pain. *Pain* (2019). https://doi.org:10.1097/j.pain.0000000000001665
5 Genschow, O., Schuler, J., Cracco, E., Brass, M. & Wänke, M. The effect of money priming on self-focus in the imitation-inhibition task: A registered report. *Experimental Psychology* **66**, 423-436 (2019).
6 Hogeveen, J. & Obhi, S. S. Altogether now: activating interdependent self-construal induces hypermotor resonance. *Cognitive neuroscience* **2**, 74-82 (2011).
7 Leighton, J., Bird, G., Orsini, C. & Heyes, C. Social attitudes modulate automatic imitation. *Journal of Experimental Social Psychology* **46**, 905-910 (2010).
8 Wang, Y. & Hamilton, A. Understanding the role of the 'self'in the social priming of mimicry. *PloS one* **8**, e60249 (2013).
9 Markus, H. R. & Kitayama, S. Culture and the self: Implications for cognition, emotion, and motivation. *Psychological Review* **98**, 224 (1991).
10 Markus, H. R. & Kitayama, S. Cultures and selves: A cycle of mutual constitution. *Perspectives on Psychological Science* **5**, 420-430 (2010).
11 Genschow, O. & Cracco, E. in *Automatic Imitation* 219-239 (Springer Nature. doi: https://doi.org/10.1007/978-3-031-62634-0_11, 2025).
12 World Bank Group. *Individuals using the Internet (% of population)*, <https://data.worldbank.org/indicator/IT.NET.USER.ZS> (2025).
13 Green, P. & MacLeod, C. J. SIMR: An R package for power analysis of generalized linear mixed models by simulation. *Methods in Ecology and Evolution* **7**, 493-498 (2016).
14 Berl, R. E. & Hewlett, B. S. Cultural variation in the use of overimitation by the Aka and Ngandu of the Congo Basin. *PloS one* **10**, e0120180 (2015).
15 Chuang, Y. C. & Fujiwara, K. Facial mimicry in unstructured dyadic interactions: A cross-cultural study. *Japanese Psychological Research* **66**, 493-509 (2024).
16 Clegg, J. M. & Legare, C. H. A cross-cultural comparison of children's imitative flexibility. *Developmental Psychology* **52**, 1435 (2016).
17 Corriveau, K. H. *et al.* Cultural differences in the imitation and transmission of inefficient actions. *Journal of Experimental Child Psychology* **161**, 1-18 (2017).
18 DiYanni, C. J., Corriveau, K. H., Kurkul, K., Nasrini, J. & Nini, D. The role of consensus and culture in children's imitation of inefficient actions. *Journal of Experimental Child Psychology* **137**, 99-110 (2015).
19 van Baaren, R. B., Maddux, W. W., Chartrand, T. L., De Bouter, C. & van Knippenberg, A. It takes two to mimic: behavioral consequences of self-construals. *Journal of personality and social psychology* **84**, 1093-1102 (2003).
20 Götz, F. M., Gosling, S. D. & Rentfrow, P. J. Small effects: The indispensable foundation for a cumulative psychological science. *Perspectives on Psychological Science* **17**, 205-215 (2022).
21 Lovakov, A. & Agadullina, E. R. Empirically derived guidelines for effect size interpretation in social psychology. *European Journal of Social Psychology* **51**, 485-504 (2021). https://doi.org:https://doi.org/10.1002/ejsp.2752

22 Faul, F., Erdfelder, E., Lang, A.-G. & Buchner, A. G* Power 3: A flexible statistical power analysis program for the social, behavioral, and biomedical sciences. *Behavior Research Methods* **39**, 175-191 (2007). https://doi.org:https://doi.org/10.3758/BF03193146

Jennifer Bellingtier, PhD
Senior Editor
Communications Psychology

Revision: Little Evidence for Cultural Differences in Automatic Imitation (COMMSPSYCHOL-25-0771B)

Dear Prof. Bellingtier,

We would like to thank you and the Reviewers for the comments we have received in response to our revision (COMMSPSYCHOL-25-0771A). We are happy that both reviewers are satisfied with our revision and recommend publication of our manuscript.

In line with your recommendations, we now follow the conventions for interpreting Bayesian evidence detailed in Schönbrodt, F.D., Wagenmakers, E. Bayes factor design analysis: Planning for compelling evidence. Psychon Bull Rev 25, 128–142 (2018).

Also, we now explain how we incorporated the additional revision instructions: https://springernature.sharepoint.com/:w:/s/NaturePortfolioERTSite/IQC6rC7ZHIPNS6rBYJeSsfuHARlITKHC0xx4kQz92ImZfnQ?rtime=me9QBazi3kg

In sum, we believe that we successfully addressed all the issues that were raised by you and hope that you share this impression. Please, let us know if there are any further issues that you would like us to address.

We are looking forward to hearing from you.

Yours sincerely,
Oliver Genschow
