# Supplementary material for: Little evidence for cultural differences in automatic imitation
Source: Commun Psychol. 2026 Oct 14;4:139. doi: 10.1038/s44271-026-00517-5 (PMC13661571; doi:10.1038/s44271-026-00517-5)
Supplement: Supplementary file 2 [file 44271_2026_517_MOESM2_ESM.pdf]

Supplement Material for

Little Evidence for Cultural Differences in Automatic Imitation

Oliver Genschow (Leuphana University Lüneburg & University of Cologne)

Mareike Westfal (Leuphana University Lüneburg & University of Cologne)

Angela R. Dorrough (FernUniversität in Hagen & University of Cologne)

Emiel Cracco (Ghent University & Leuphana University Lüneburg)

Tobias Wingen (FernUniversität in Hagen)

Jan Crusius (University of Greifswald)

Experiment 1

Error Rate Analysis

A 2 (congruency: congruent vs. incongruent trials) x 2 (nation: USA vs. Vietnam) mixed ANOVA on participant's error rates with nation as between- and congruency as within-subjects factor yielded a significant main effect of congruency, $F(1, 406) = 71.36$, $p < .001$, $\eta_p^2 = .15$, 95% CI [.10, .20], $BF_{01} < 0.001$, indicating that participants made fewer errors in congruent trials ($M = 1.78$ %; $SD = 2.25$) than in incongruent trials ($M = 2.87$ %; $SD = 2.85$). There was also a significant interaction between congruency and nation, $F(1, 406) = 16.61$, $p < .001$, $\eta_p^2 = .04$, 95% CI [.01, .07], $BF_{01} = 0.002$, indicating larger congruency effects for people living in Vietnam ($M = 1.60$ $SD = 2.84$) than for people living in the US ($M = 0.56$; $SD = 2.28$). The main effect for nation did not reach conventional levels of significance, $F(1, 406) = 3.73$, $p = .054$, $\eta_p^2 = .01$, 95% CI [.000, .04], $BF_{01} < 0.007$.

Controlling for Age

In an additional reaction time based analysis, we accounted for potential age-related variability by including age as a covariate in the mixed model analysis reported in the main manuscript. Because data were collected in only two nations, nation was included as a fixed effect. In this model, the overall effects of congruency, ($B = 19.99$, 95% CI[8.56, 31.42], $SE = 5.83$, $p < .001$, $BF_{01} = 0.057$) and nation, ($B = -94.75$, CI[-125.94, -63.55], $SE = 15.94$, $p < .001$, $BF_{01} < .001$) remained significant. Additionally, there was a significant main effect of age ($B = -1.74$, 95% CI[-2.97, -0.52], $SE = 0.63$, $p = .006$, $BF_{01} = 0.400$). However, the interaction between trial and nation, ($B = 3.75$, 95% CI[-3.43, 10.93], $SE = 3.66$, $p = .306$, $BF_{01} = 10.68$) remained insignificant even after controlling for age.

Experiment 2

Additional Control Checks

We checked whether there is a difference between cultures in terms of internet usage by analyzing internet usage data from the year 2023[1]—the year in which we conducted

Experiment 2. There was no statistically meaningful difference in the percent of the population using the internet between collectivistic (*M* = 87.00 %; *SD* = 8.98) and individualistic cultures (*M* = 92.55 %; *SD* = 4.30), $t(20) = 1.85$, $p = .079$, $d = .79$, 95% CI [-0.71, 11.81]. A Bayes Factor computed in JASP with the default priors (i.e., Cauchy prior width) of 0.707 to test for evidence that there is a difference between the cultures yielded $BF_{10} = 1.241$ indicating that there is not much evidence for the idea that the two cultures differ in terms of internet usage.

Supplementary Figure S1. *Geographic Location and Individualism/Collectivism Scores of Investigated Nations*

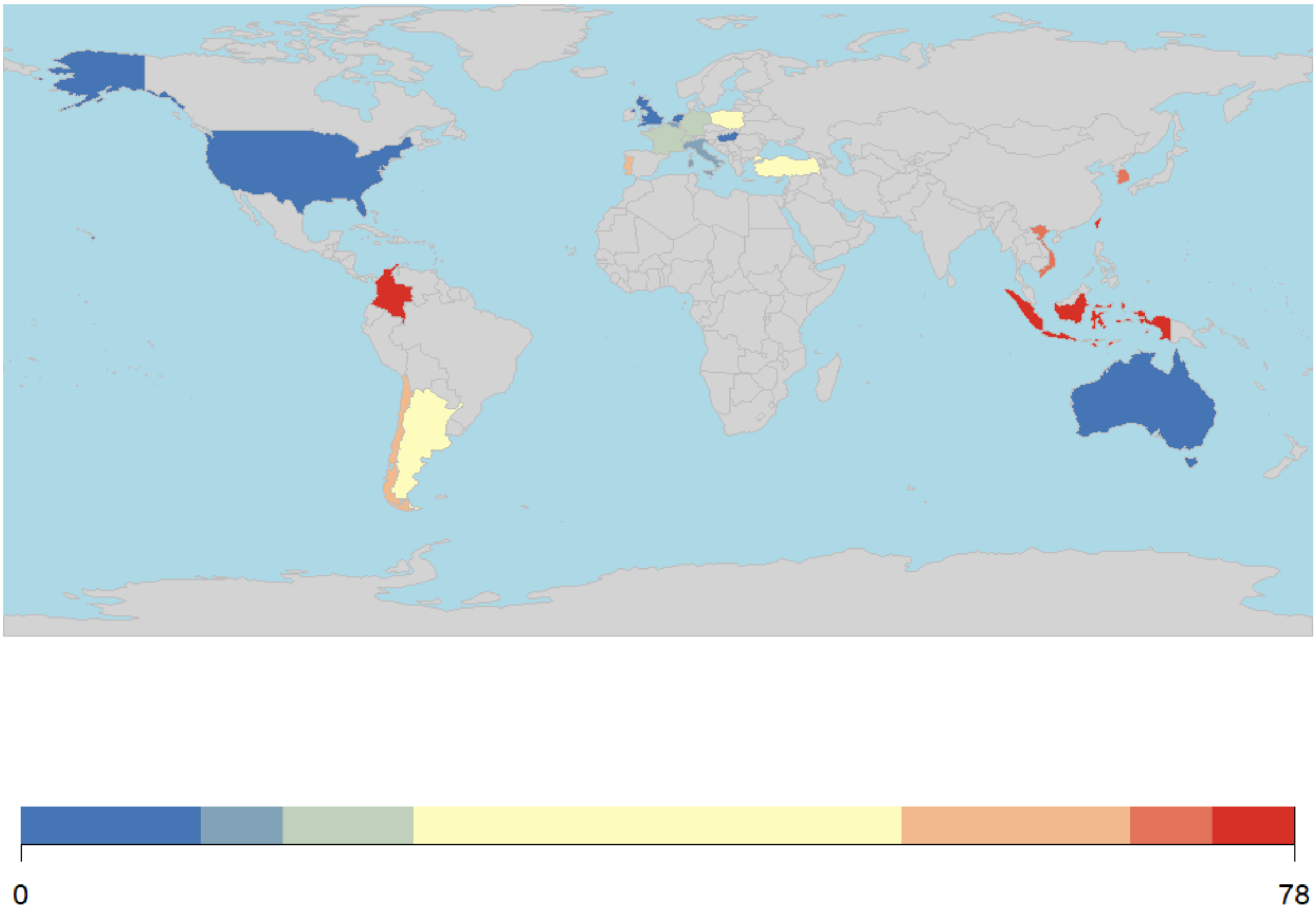

Note. Colors indicate the nations' individualism/collectivism, ranging from blue (minimal collectivism) to red (maximal collectivism).

Supplementary Figure S2. *Random Intercepts and Slopes Across Nations on Participants' Reaction Times*

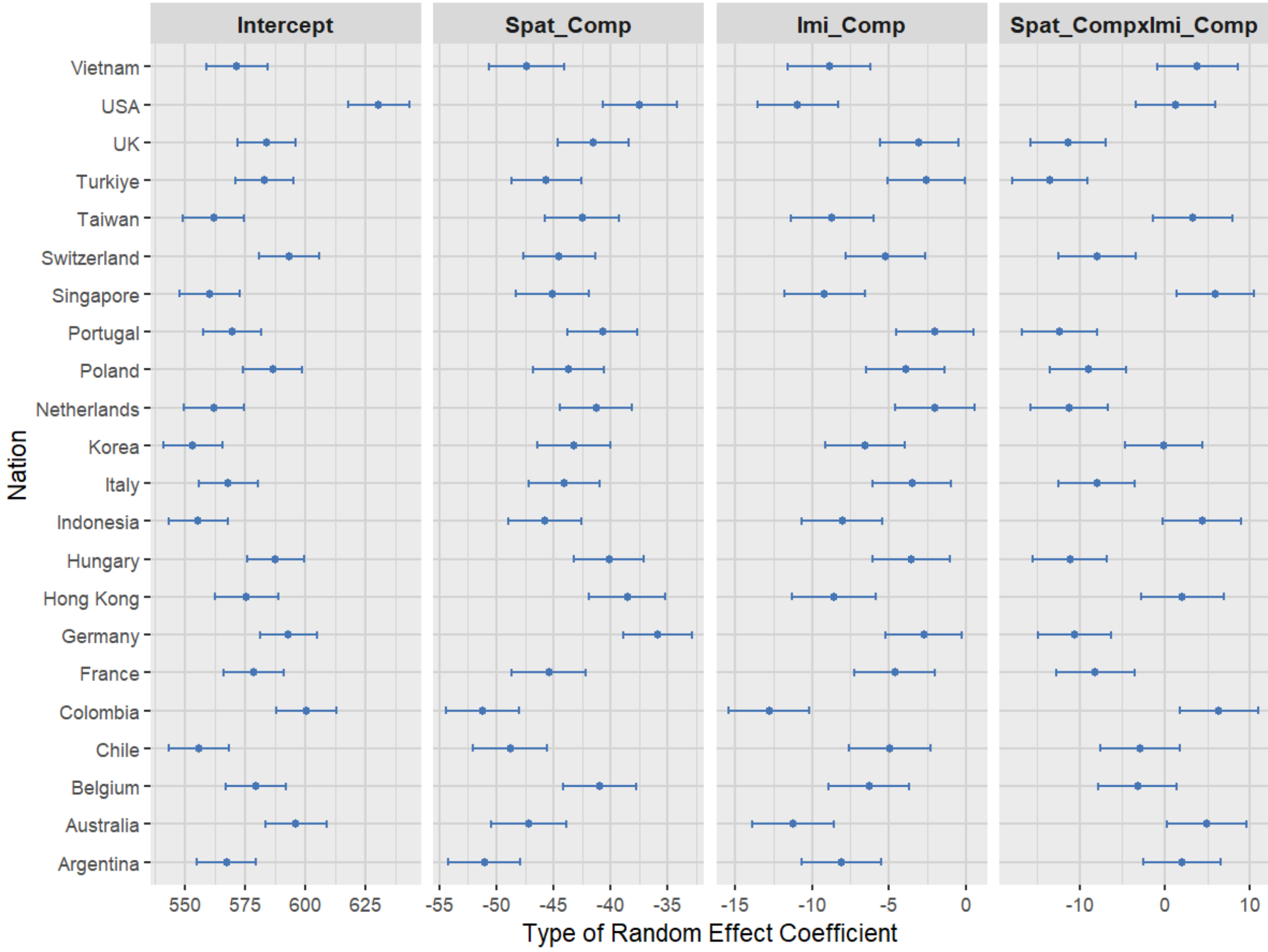

Note. Model coefficients from a mixed model with random intercepts and random slopes presented with 95% Confidence Intervals (*N* = 4,447 participants). "Spat_Comp" represents spatial compatibility, "Imi_Comp" represents imitative compatibility, and the rightmost panel represents the interaction terms.

Controlling for age

To ensure that our primary findings were not driven by age-related differences between nations, we repeated all main mixed-model analyses from Experiment 2 while including age as a covariate[a]. Overall, controlling for age did not change the pattern of our results, and all key effects remained significant.

First, we repeated the initial mixed model investigating the influences of spatial and imitative compatibility on reaction times (including random intercepts for participants and nations), this time adding age as a continuous predictor. While age was a significant covariate (B = 1.32, 95% CI[1.17, 1.47], SE = 0.08, p < .001, BF_{01} < 0.001), the main effects of spatial compatibility (B = −43.73, 95% CI[-44.46, -42.99], SE = 0.38, p < .001, BF_{01} < .001) and imitative compatibility (B = −6.12, 95% CI[-6.87, -5.38], SE = 0.38, p < .001, BF_{01} < 0.001) remained highly significant. Importantly, the interaction between spatial and imitative compatibility also remained robust, B = −3.71, 95% CI[-4.76, -2.67], SE = 0.53, p < .001, BF_{01} < 0.001.

Next, we repeated the model that accounted for nation-level variability by including random slopes. Again, controlling for age (B = 1.32, 95% CI[1.17, 1.47], SE = 0.08, p < .001, BF_{01} < 0.001) did not alter the core findings. The overall effects of spatial compatibility (B = −43.79, 95% CI[-45.75, -41.84], SE = 1.00, p < .001, BF_{01} < 0.001), imitative compatibility (B = −6.27,95% CI[-7.90, -4.63], SE = 0.83, p < .001, BF^{01} < 0.001), and their interaction (B = −3.42, 95% CI[-6.64, -0.20], SE = 1.64, p = .049, BF_{01} = 0.739) all remained significant.

Finally, we re-tested the relationship between these compatibility effects and culture by including collectivism and its interaction terms in the model, while controlling for age. Consistent with our main analyses, neither the interaction between imitative compatibility and collectivism (B = −0.04, 95% CI[-0.10, 0.02], SE = 0.03, p = .173, BF_{01} = 1.86) nor the

[a] When merging the age and experimental datasets, 0.34% of the trial data could not be matched and was coded as missing.

interaction between spatial compatibility and collectivism (*B* = −0.06, 95% CI[-0.12, 0.01], *SE* = 0.03, *p* = .094, BF_{01} = 1.19) reached significance. Crucially, the focal three-way interaction between spatial compatibility, imitative compatibility, and collectivism remained significant in this age-controlled model, *B* = 0.12, 95% CI[0.02, 0.23], *SE* = 0.05, *p* = .027, BF_{01} = 0.453.

Error Rates Analysis

We repeated the analyses we conducted on reaction times, now focusing on participants' error rates. As for reaction times, we first investigated the effects of spatial compatibility and imitative compatibility on error rates in a mixed model including random intercepts for participant and nation (to account for the repeated-measurement design). This analysis yielded significant effects for both spatial compatibility, B = -0.047, 95% CI[-0.048, -0.045], SE < 0.001, p < .001, and imitative compatibility, *B* = −0.010, 95% CI[-0.012, -0.008], *SE* < 0.001, *p* < .001. These results indicate that participants made fewer errors in both types of compatible trials. Moreover, the interaction between spatial and imitative compatibility was also significant, *B* = 0.006, 95% CI[0.004, 0,008], *SE* = 0.001, *p* < .001 indicating that the effect of imitative compatibility was reduced under high spatial compatibility (see Supplementary Figure 3 for details).

In the next step, we accounted for potential nation-level variability by including random slopes in the model. In this model, the overall effects of spatial compatibility, *B* = −0.047, 95% CI[-0.051, -0.043], *SE* = 0.002, *p* < .001, imitative compatibility, *B* = −0.010, 95% CI[-0.012, -0.082], *SE* < 0.001, *p* < .001, and the interaction, *B* = 0.006, 95% CI[0.003, 0.009], *SE* = 0.001, *p* < .001, all remained significant.

While we observed some variation across nations, overall, the coefficients were significant in all nations (as indicated by all CIs excluding zero) and in the same direction (see Supplementary Figure 4).

Supplementary Figure S3. *Error Rates as a Function of Compatibility*

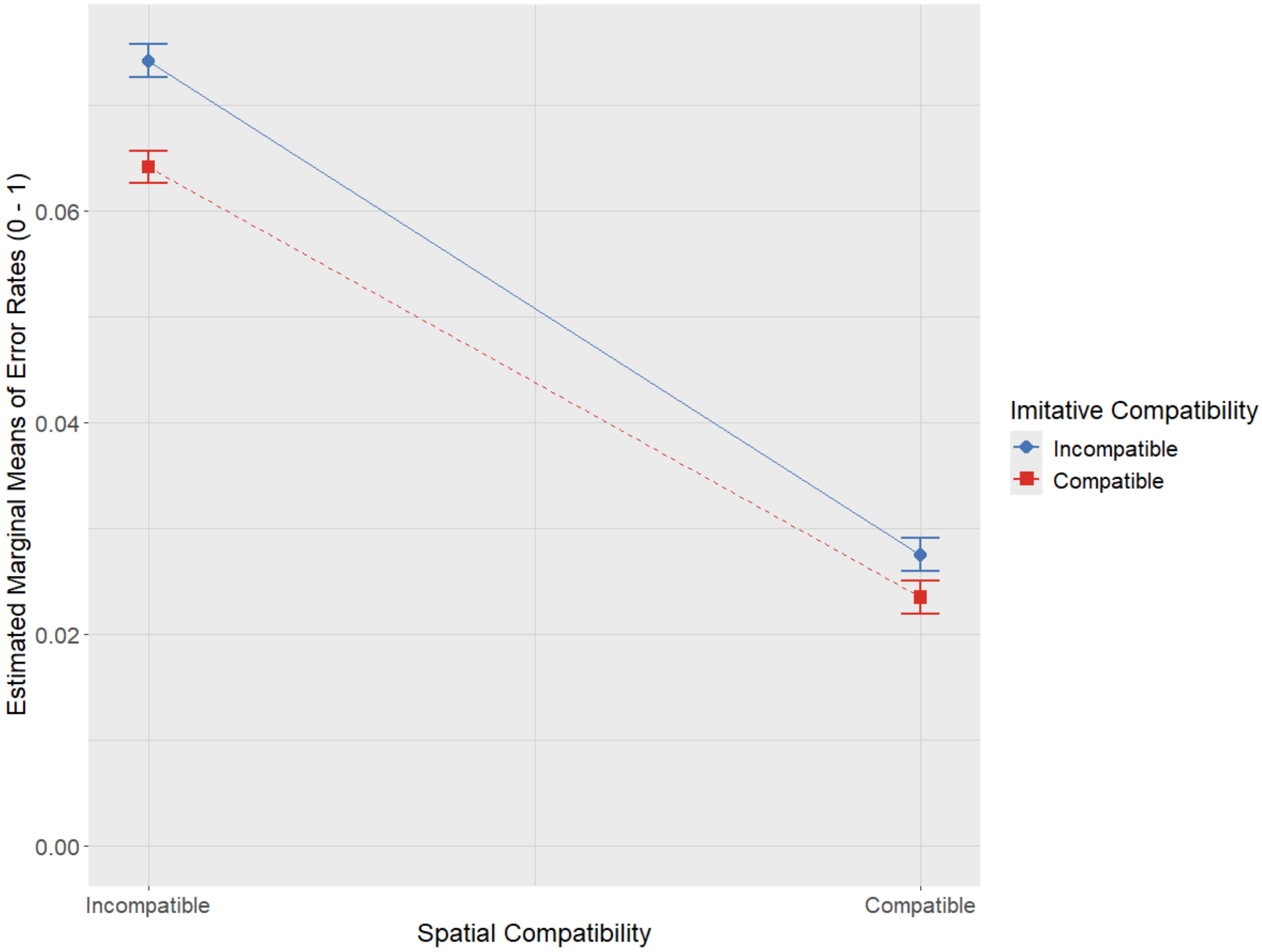

Note. Error Rates (N = 4,447 participants) are calculated as average of correct (0) and incorrect (1) trials (i.e., a value of 0 would indicate 100% correct trials). Error bars represent the standard errors.

In line with these overall consistent results across nations, we also observed no significant three-way interaction between spatial and imitative compatibility and collectivism, $B < 0.001$, 95% CI[-0.00001, 0.0002], $SE < 0.001$, $p = .109$.

Supplementary Figure S4

Random Intercepts and Slopes for Predicting Error Rates Across Nations

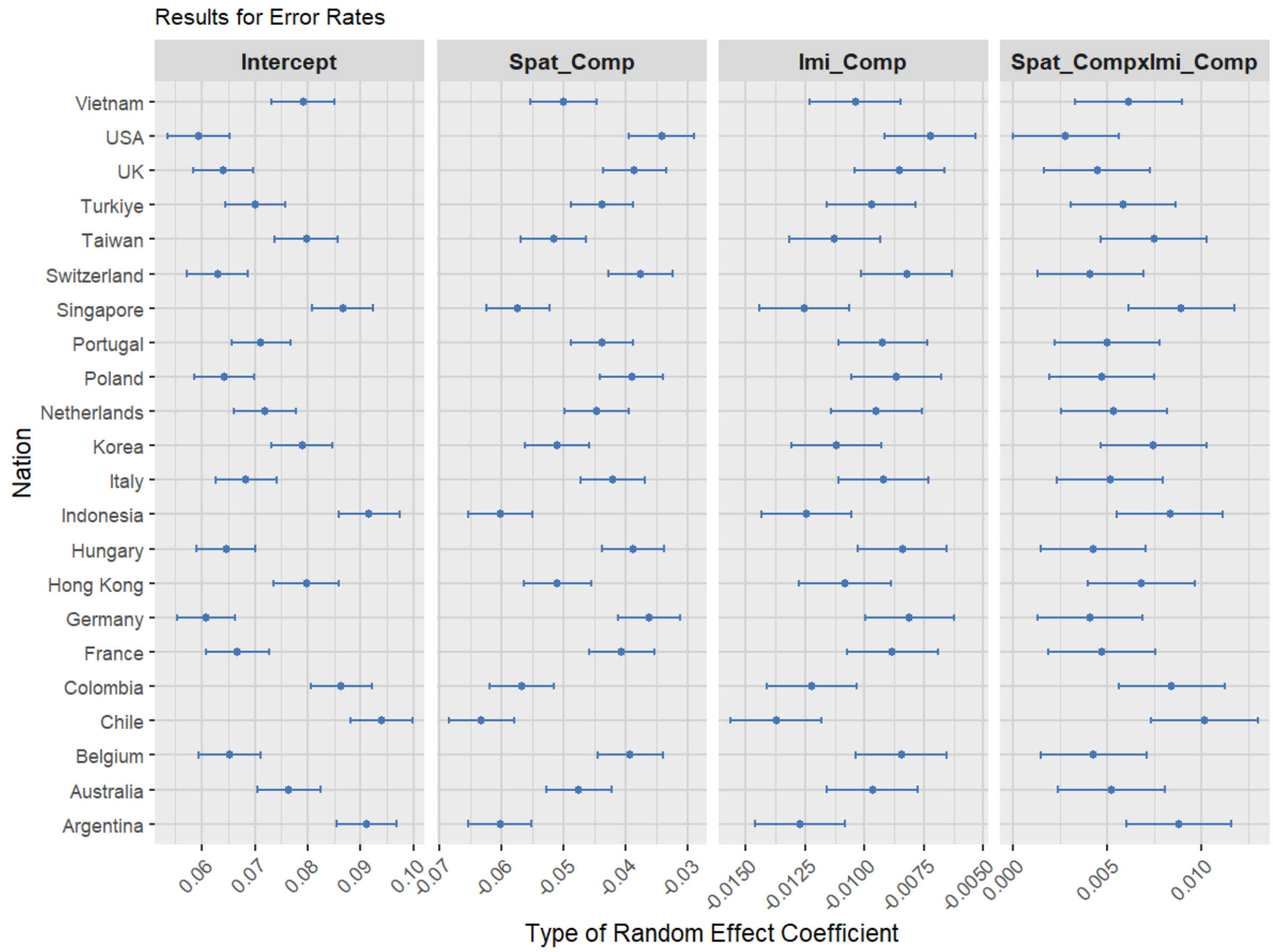

Note. Model coefficients from a mixed model with random intercepts and random slopes predicting error rates, presented with 95% Confidence Intervals (*N* = 4,447 participants). "Spat_Comp" represents spatial compatibility, "Imi_Comp" represents imitative compatibility, and the rightmost panel represents the interaction terms. Negative values represent fewer errors.

References

1 World Bank Group. *Individuals using the Internet (% of population)*, <https://data.worldbank.org/indicator/IT.NET.USER.ZS> (2025).
